# Enhancement of the anticancer effect of atorvastatin-loaded nanoemulsions by improving oral absorption via multivalent intestinal transporter-targeting lipids

**DOI:** 10.1080/10717544.2022.2149896

**Published:** 2022-11-23

**Authors:** Laxman Subedi, Prashant Pandey, Bikram Khadka, Jung-Hyun Shim, Seung-Sik Cho, Seho Kweon, Youngro Byun, Ki-Taek Kim, Jin Woo Park

**Affiliations:** a Department of Biomedicine, Health & Life Convergence Sciences, BK21 Four, Biomedical and Healthcare Research Institute, Mokpo National University, Jeonnam, Republic of Korea; b College of Pharmacy and Natural Medicine Research Institute, Mokpo National University, Jeonnam, Republic of Korea; c Department of Molecular Medicine and Biopharmaceutical Science, Graduate School of Convergence Science and Technology, College of Pharmacy, Seoul National University, Seoul, Republic of Korea; d Research Institute of Pharmaceutical Sciences, College of Pharmacy, Seoul National University, Seoul, Republic of Korea

**Keywords:** Atorvastatin, nanoemulsion, bile acid-mediated uptake, multivitamin-facilitated transport, oral bioavailability, oral chemotherapy

## Abstract

Atorvastatin (ATV) has attracted considerable attention as a potential therapeutic agent for cancer because it inhibits cancer cell proliferation by suppressing the mevalonate pathway. However, because of its low oral absorption, high doses of ATV are required for chemotherapeutic applications. In this study, we constructed ATV-loaded nanoemulsions (ATV-NEs) containing multivalent intestinal transporter-targeting lipids to improve the oral bioavailability of ATV. ATV-NEs were prepared via oil-in-water emulsification for transporter-targeted delivery, and contained the following anchors: an ionic complex of deoxycholic acid (DOCA) with the cationic lipid 1,2-dioleyl-3-trimethylammonium propane (DOTAP) (DOCA-DOTAP), a biotin-conjugated lipid (Biotinyl PE), and d-alpha-tocopherol polyethylene glycol succinate (TPGS) to allow bile acid- and multivitamin transporter-mediated permeation of ATV without P-glycoprotein (P-gp)-mediated efflux. The optimized formulation (ATV-NE#6) had 1,091% higher oral bioavailability than free ATV. Finally, treatment of 4T1 cell-bearing mice with oral ATV-NE#6 (equivalent to 40 mg/kg ATV) significantly suppressed tumor growth; the maximum tumor growth reduction was 2.44-fold that of the control group. The results thus suggest that ATV-NEs allow for effective oral chemotherapy by enhancing the oral bioavailability of ATV.

## 1. Introduction

Statins, 3-hydroxy-3-methylglutaryl coenzyme A (HMG-CoA) reductase inhibitors, are widely used to control hypercholesterolemia (Maron et al., 2000). HMG-CoA reductase is the rate-limiting enzyme in mevalonate synthesis, which is the precursor for cholesterol biosynthesis (Endo et al., 1977). This pathway also produces numerous isoprenoid derivatives that are critical for cell growth, differentiation, migration, and intracellular trafficking (Gazzerro et al., 2012, Pisanti et al., 2014). Transformed malignant cells are also highly dependent on the mevalonate pathway for the synthesis of lipid moieties that are critical for cell proliferation, membrane integrity, cell cycle progression, and cell signaling (He et al., 2015). Thus, the statin-mediated inhibition of HMG-CoA reductase decreases the levels of mevalonate and its downstream products, which have important roles in multiple cell functions, including membrane integrity, cell signaling, protein synthesis, and cell cycle progression; this inhibition of HMG-CoA results in significant inhibition of cancer cell growth (Brown & Goldstein, 1974; Buhaescu & Izzedine, 2007). Moreover, the level of HMG-CoA reductase is associated with carcinogenesis and tumor progression in several types of cancer. The use of statins to target HMG-CoA reductase can increase tumor-specific apoptosis, induce cell cycle arrest, and inhibit tumor growth (Nishida et al., 2005; Bjarnadottir et al., 2015; Sethunath et al., 2019). Numerous *in vitro* and *in vivo* studies have confirmed that the mechanisms underlying these anticancer effects involve suppression of tumor growth, induction of apoptosis and autophagy, inhibition of cell migration and invasion, and inhibition of angiogenesis (Martínez-Botas et al., 2001; Fuchs et al., 2008; Martinez et al., 2018; Sheng et al., 2020; Wang et al., 2020).

Atorvastatin (ATV) is a lipophilic synthetic statin commonly used for the management of lipid levels; it has greater beneficial effects and fewer side effects, compared with simvastatin (Wierzbicki et al., 1999). ATV is also associated with decreased risks of recurrence and mortality in cancer. ATV reportedly can modulate cell apoptosis, antiproliferative effects, cell growth inhibition, and cell cycle arrest (Xiao et al., 2008). Thus, it can effectively inhibit tumor growth in breast, prostate, pancreatic, and liver cancer. Furthermore, it can inhibit ovarian cancer cell proliferation by inducing apoptosis, autophagy, cellular stress, and cell cycle G1 arrest through the inhibition of AKT/mTOR and activation of the mitogen-activated protein kinase (MAPK) pathways (Jones et al., 2017). It can also inhibit cancer cell adhesion and invasion while decreasing the expression levels of vascular endothelial growth factor (VEGF) and matrix metallopeptidase-9 (MMP-9) through the downregulation of c-Myc in ovarian cancer cells; additionally, it can inhibit breast cancer proliferation by downregulating the phosphatase and tensin homologue (PTEN)/AKT pathway via promotion of the expression of Ras homolog family member B (RhoB) (Chen et al., 2012). Finally, it can induce autophagy in hepatocellular carcinoma and colorectal carcinoma cells by activating protein kinase (AMPK)/p21 signaling and stimulating the endoplasmic reticulum (ER) stress response (Ma et al., 2019). Moreover, ATV shows synergistic antiproliferative, antiangiogenic, and apoptotic actions against various types of cancers (e.g., colon, lung, and prostate), in combination with anticancer agents such as doxorubicin, cisplatin, paclitaxel, topotecan, bevacizumab, and celecoxib (Zheng et al., 2010; Buranrat et al., 2017; Tan et al., 2017; Ma et al., 2019; Guo et al., 2020; Lee et al., 2021; Marti et al., 2021). Therefore, ATV has received considerable interest as a potential therapeutic agent for use in cancer treatment.

Although the anticancer activities of statins have been extensively investigated, the *in vivo* and clinical effects have been highly dependent on their physiochemical properties, such as lipophilicity, dose, and treatment period (Longo et al., 2020). Furthermore, several studies suggested that high doses, sometimes > 100-fold greater than doses used in the treatment of hypercholesterolemia, are necessary to achieve optimal anticancer efficacy (Dulak & Józkowicz, 2005; Jiang et al., 2014). However, their therapeutic anticancer effects are limited by low oral bioavailability (< 14%) due to poor aqueous solubility, as well as pre-systemic clearance by the gastrointestinal (GI) mucosa and first-pass metabolism in the liver (Khan & Dehghan, 2011). Therefore, it is important to improve the intestinal absorption capacity and systemic availability of these low-permeability drugs.

Most approaches to improve the oral absorption of ATV have mainly focused on increasing its solubility by incorporation into nanoparticle systems, such as nanoemulsions (NEs), micelles, liposomes, solid lipid nanoparticles, and lecithin nanoparticles (Tiwari & Pathak, 2011; Shamsuddin et al., 2016; Elmowafy et al., 2017; Xie et al., 2017; Sarangi et al., 2018) . Encapsulation in nanoparticles can effectively increase the intestinal supersaturation concentration, enhance the physiological stability, and facilitate the transepithelial transport of poorly available drugs through endocytosis by enterocytes (Jain et al., 2013). Furthermore, surfactants and co-surfactants composed of nanocarriers can promote drug transport across the intestinal epithelia by reversibly opening tight junctions, while also altering the integrity and/or increasing the fluidity of the epithelial membrane (Pangeni et al., 2021). However, most enhancers of oral delivery are mild and nonionic surfactant-like materials that must be present in large amounts in the formulations to minimize the dilution and spreading effects in the GI tract (Maher et al., 2019).

Alternatively, for enhanced intestinal permeation, the surface modification of nanocarriers with various moieties targeting intestinal membrane transporters (e.g., peptide transporter, glucose transporter, apical sodium-dependent bile acid transporter [ASBT], and sodium-dependent multivitamin transporter [SMVT]) has been introduced to facilitate drug transport (Pangeni et al., 2021). In our previous study, we improved the oral absorption of poorly permeable drugs by ionic complex formation with a positively charged bile acid derivative via ASBT-mediated transport (Deng & Bae, 2020; Li et al., 2020; Pangeni et al., 2020; Subedi et al., 2022). Additionally, we constructed docetaxel- and etoposide-loaded nanoemulsive systems to increase aqueous solubility and intestinal permeability by physically anchoring deoxycholic acid (DOCA) in an ionic complex with the cationic lipid, 1,2-dioleyl-3-trimethylammonium propane (DOTAP) (DOCA-DOTAP); we confirmed 249% and 1752% greater oral bioavailabilities, respectively, compared to the free drugs via ASBT-mediated endocytosis (Jha et al., 2020a; Jha et al., 2020b)

The main objective of this study was to design a nanoemulsive system for ATV to improve its effectiveness for oral cancer treatment by enhancing aqueous solubility and intestinal membrane permeability via ASBT- and SMVT-facilitated transport, along with the suppression of P-glycoprotein (P-gp)-mediated efflux. For this purpose, ATV was incorporated into oil-in-water (o/w) NEs (ATV-NEs); then, the DOCA-TAP, biotin-conjugated lipid (Biotinyl PE), and d-alpha-tocopherol polyethylene glycol succinate (TPGS) were physically anchored onto the nanodroplets for targeting of multiple transporters (i.e., ASBT, SMVT, and P-pg). Next, to confirm the increase in oral absorption of ATV-NEs, their permeabilities were evaluated across artificial membranes and Caco-2/HT-29 MTX E12 cell monolayers. Furthermore, their intestinal transport mechanisms, which involve the targeting of multiple transporters in combination with other pathways, were investigated by using inhibitors for distinct cellular uptake pathways. Finally, the *in vitro* cytotoxic effects and antimetastatic efficacy of ATV-NEs were evaluated in a breast cancer cell line, the oral bioavailability of ATV-NEs was evaluated in rats, and the antitumor efficacy of oral ATV-NEs was evaluated in 4T1 cell tumor-bearing mice.

## 2. Experimental design

### 2.1. Materials

ATV and simvastatin as an internal standard (IS) for ATV were obtained from Shangyu Jingxin Pharmaceutical Co., Ltd. (Weisan, Zhejiang, China). Propylene glycol monocaprylate type II (Capryol 90), polyoxyethylene sorbitan monooleate 80 (Tween 80), TPGS, sodium DOCA, methyl-β-cyclodextrin (MBCD), genistein, actinomycin D (Act D), chlorpromazine, cyclosporine A (Cys A), brefeldin A, ethylene glycol-bis-(2-aminoethyl ether)-N,N,N′,N′-tetraacetic acid (EGTA), hemi-calcium salt of pantothenic acid (PA), and clofazimine (CFZ) were purchased from Sigma-Aldrich, Inc. (St. Louis, MO, USA). Diethylene glycol monoethyl ether (Transcutol HP) was provided by Gattefossé (Saint Priest, France). 1,2-Dioleoyl-sn-glycero-3-phosphoethanolamine-*N*-(biotinyl) (sodium salt) (Biotinyl PE) and 1,2-dioleyl-3-trimethylammonium propane (DOTAP) were obtained from Avanti Polar Lipids (Alabaster, AL, USA). Other chemicals for high-performance liquid chromatography (HPLC) and ultra-performance liquid chromatography-tandem mass spectrometry (UPLC-MS/MS) analyses were obtained from Thermo Fisher Scientific, Inc. (Waltham, MA, USA) and Alfa Aesar (Ward Hill, MA, USA), respectively. All chemical reagents used in this study were of analytical grade.

### 2.2. Animals

Sprague–Dawley rats (male, 6 weeks old, 220 g) and BALB/c mice (female, 6 weeks old, 20 g) were purchased from G-bio (Gwangju, Republic of Korea). Ethical approval for the animal experiments was obtained from the Institutional Animal Care and Use Committee of Mokpo National University (Jeonnam, Republic of Korea; approval nos. MNU-IACUC-2021-018 [September 23, 2021] and MNU-IACUC-2022-003 [March 2, 2022]). All animal experiments were performed in accordance with the National Institutes of Health Guidelines for the Care and Use of Laboratory Animals and the guidelines of the Institutional Animal Care and Use Committee.

### 2.3. Preparation and characterization of ATV-NEs

**Figure 1.**
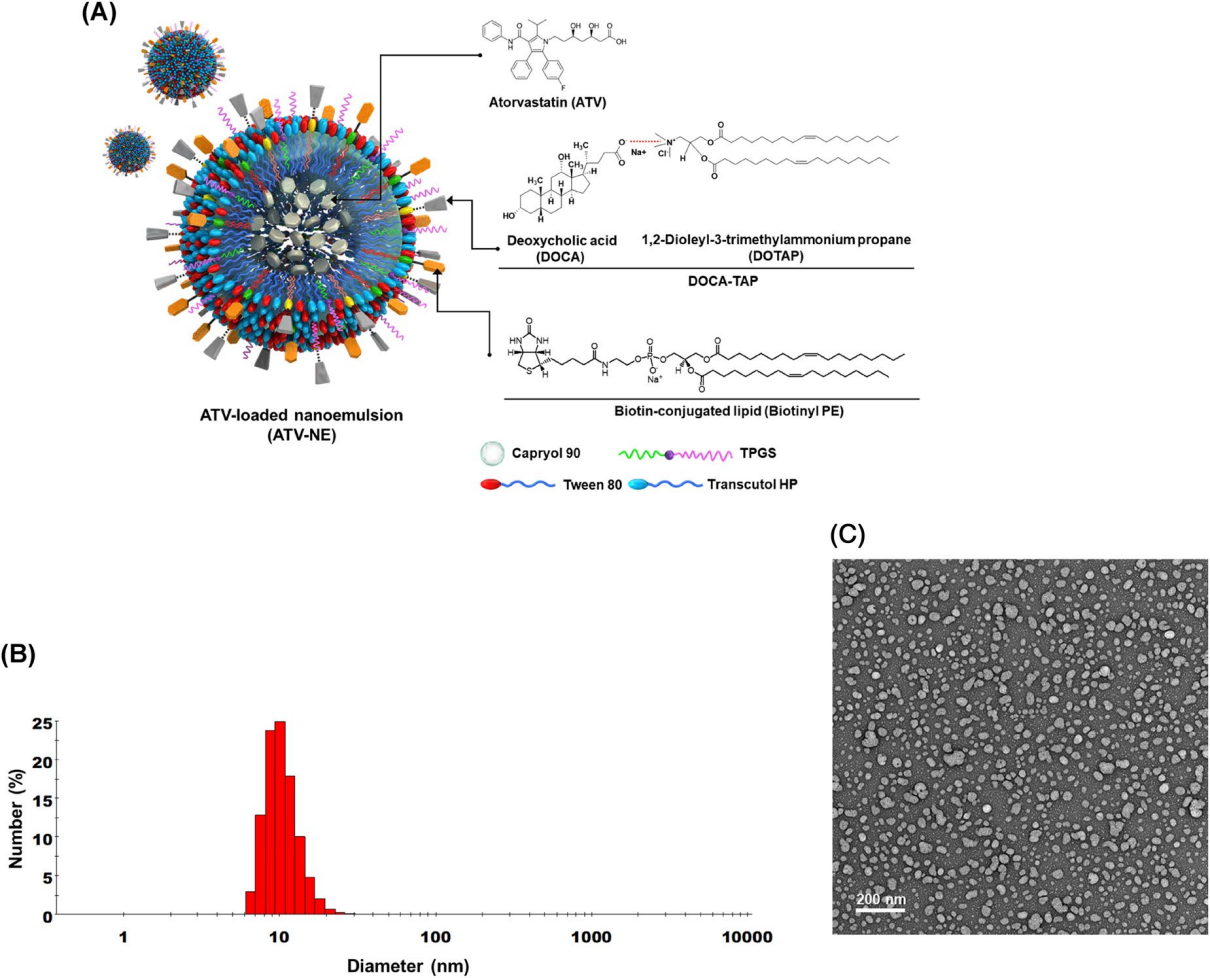
Preparation and characterization of ATV-NEs. (A) Schematic illustration of the ATV-loaded nanoemulsions (ATV-NEs) with anchoring of DOCA-TAP and Biotinyl PE. (B) Particle size and (C) transmission electron micrograph of ATV-NE#6. Scale bar: 200 nm.

To obtain stable ATV-loaded NEs (ATV-NEs), 10 mg of ATV were dispersed in 42 mg of oil (Capryol 90). Subsequently, 550 mg of a mixture of surfactant and co-surfactant (S_mix_; Tween 80:Transcutol HP, 1:2 [w/w]), and 50 mg TPGS were added to form a self-microemulsifying drug delivery system (Ishak & Osman, 2015; Jha et al., 2020a). Furthermore, to examine the improvement in permeability of ATV via ASBT- and SMVT-mediated transport, DOTAP was integrated with DOCA at a molar ratio of 1:1 by ionic complexation to form DOCA-TAP, as described previously (Jha et al., 2020a). Then, effective enhancers, such as DOCA-TAP and/or Biotinyl PE, were anchored onto the S_mix_ layer of ATV-NEs using a low-energy spontaneous emulsification method. Separately, DOTAP or DOCA was incorporated into the ATV-NEs to confirm the anchoring of DOCA-TAP onto the S_mix_ (Figure 1(A), Table 1).

**Table 1.** Compositions of ATV-NEs.

Formulation code	ATV (mg)	Capryol 90 (mg)	Tween 80 (mg)	Transcutol HP (mg)	TPGS (mg)	DOTAP (mg)	DOCA (mg)	DOCA-TAP (mg)	Biotinyl PE (mg)
ATV-NE#1	10	42	250	500	50				
ATV-NE#2	10	42	250	500	50	16.8			
ATV-NE#3	10	42	250	500	50		10		
ATV-NE#4	10	42	250	500	50			26.8	
ATV-NE#5	10	42	250	500	50				11.92
ATV-NE#6	10	42	250	500	50			26.8	11.92

To characterize the ATV-NEs, particle size, polydispersity index, and zeta potential were examined using a dynamic laser light scattering analyzer (Malvern Zetasizer Nano ZS90: Malvern Instruments, Malvern, UK) after ATV-NEs had been diluted 1:10 with deionized water and sonicated for 1 min to minimize multiple scattering effects. Additionally, transmission electron microscopy was performed for morphological evaluation of selected NEs. Briefly, a drop of NEs diluted with water was placed on the surface of a copper grid, negatively stained with an aqueous solution of 2% phosphotungstic acid, and observed by transmission electron microscopy (JEM-200; JEOL, Tokyo, Japan).

### 2.4. In vitro artificial intestinal membrane permeability

An *in vitro* artificial intestinal membrane permeability study of ATV-NEs was performed using precoated 96-well plates specifically designed for this assay (BD Biosciences, San Jose, CA, USA). First, 300 µL of phosphate-buffered saline (PBS, pH 6.8) were added to each well of the acceptor plate and then assembled with the donor plate. Next, ATV-NEs were further diluted with PBS (pH 6.8) to 100 µg/mL ATV; 200 µL of the sample solution were then loaded into each well of the donor plate. After incubation at room temperature for 5 h, the samples from both donor and acceptor plates were collected and ATV in the collected samples was analyzed by HPLC with a C18 column (4.6 × 250 mm, 5 µm, 100 Å, 50 µL sample injection) The flow rate of the mobile phase (0.01 M sodium dihydrogen phosphate:acetonitrile, 60:40 [v/v]) was 1 mL/min, and ATV was detected using an ultraviolet-visible detector at a wavelength of 254 nm. The effective permeability (*P_e_*) of ATV was calculated by the following formula:

(1) $$P_{e}=-\;\ln[1\;-{\;C}_{A}\text{(t)}/C_{\text{eq}}]/[S_{e}\times \;(1/V_{D}+1/V_{A})\times \;t]$$

where *P_e_* is the permeability (cm/s), S_e_ is the effective permeation area (0.288 cm^2^), V_D_ is the volume of the donor compartment (0.2 mL), V_A_ is the volume of the acceptor compartment (0.3 mL), t is the total incubation time in seconds, C_A_(t) is the drug concentration in the acceptor well at time t, and C_eq_ represents [C_D_(t) × V_D_ + C_A_(t) × V_A_]/(V_D_ + V_A_), where C_D_(t) denotes the drug concentration in the donor well at time t.

### 2.5. In vitro permeability across a Caco-2/HT-29 MTX E12 cell monolayer

To evaluate the apparent permeability (*P_app_*) of different formulations of ATV-NEs across a monolayer composed of Caco-2 and HT-29 MTX E12 cells, Caco-2 (ATCC^®^ HTB-37™; American Type Culture Collection, Manassas, VA, USA) and HT-29 MTX E12 (EACC 12040401; Public Health England, Oxford, UK) cells were mixed at a ratio of 8:2 (based on cell number) and seeded onto 24-well Transwell^®^ filter inserts at density of 1 × 10^5^ cells/well. After the cells had formed a complete monolayer with a transepithelial electrical resistance (TEER) value of 450 ± 100 Ω·cm^2^, the medium in each well was replaced with prewarmed Hank’s balanced salt solution (HBSS) and allowed to equilibrate for 30 min. Then, HBSS in the receptor chamber was replaced with 0.6 mL of fresh HBSS solution; 0.1 mL of each sample solution in HBSS equivalent to 100 µg/mL ATV was added to the apical region of the donor chamber, and the cells were incubated at 37 °C. At predetermined time points (0, 0.5, 1, 2, 3, 4, and 5 h), 0.2-mL aliquots of the sample in the receptor chamber were withdrawn and replaced with the same volume of HBSS. The collected samples were filtered with 0.45-μm polyvinylidene fluoride filters and the permeated ATV was analyzed by HPLC at a wavelength of 254 nm, as described above.

The *P_app_* for ATV in various formulations was calculated as follows:

(2) $$P_{\mathrm{app}}={\text{dM}}_{a}/\text{dt}\times 1/S_{e}\times C_{0}$$

where dM_a_/dt is the linear appearance rate of mass on the acceptor region (μg/s), S_e_ is the surface area of the monolayer (0.33 cm^2^), and C_0_ is the initial concentration of ATV in the donor compartment (μg/mL).

### 2.6. In vitro cellular uptake in Caco-2 and MDCK cells

#### 2.6.1. Intracellular uptake by Caco-2 and ASBT-transfected MDCK cells

To investigate the enhanced cellular uptake of ATV via ASBT- and SMVT-facilitated transport of ATV-NEs, Caco-2 cells were seeded onto coverslips coated with Cell-Tak cell and tissue adhesive (Corning, Inc., Corning, NY, USA) and cultured in Dulbecco’s Modified Eagle’s Medium supplemented with 10% (v/v) fetal bovine serum (FBS) and 1% penicillin/streptomycin until they formed a monolayer. After cells had been treated for 1 h with coumarin 6 and ATV dispersed in water or DMSO, or coumarin 6-coloaded-ATV-NE#1, ATV-NE#3, ATV-NE#4, ATV-NE#5, and ATV-NE#6 at a concentration equivalent to 100 µg/mL ATV, the cells were washed with ice-cold PBS (pH 7.4) and fixed with 4% formaldehyde in PBS (pH 7.4). Then, the actin filaments and nuclei of the cells were stained with phalloidin-rhodamine (100 nM) and 4′,6-diamidine-2′-phenylindole dihydrochloride (DAPI), respectively. The coverslips were washed with ice-cold PBS (pH 7.4) and the cellular uptake was examined by confocal laser scanning microscopy (Carl Zeiss, Oberkochen, Germany).

After DOCA-TAP and/or Biotinyl PE had been anchored onto the NEs, the enhancement of intestinal permeability of ATV from the ATV-NEs (via interactions with bile acid transporters and multivitamin transporters) was evaluated using human ASBT gene-transfected Mardin–Darby canine kidney (MDCK) cells (ATCC^®^ CCL-34™) in the presence or absence of SMVT inhibitor (i.e., PA). Briefly, MDCK cells were seeded at a density of 1 × 10^4^ cells/coverslip and then transfected with the human ASBT gene (SLC10A2 human cDNA open reading frame [ORF] clone; OriGene Technologies, Inc., Rockville, MD, USA) by using Lipofectamine 2000^®^ and P300™ (Thermo Fisher Scientific). After the formation of a confluent monolayer, the cells were treated with 100 µL of coumarin 6 and ATV dispersed in water or 0.5% DMSO, or coumarin 6-coloaded ATV-NE#1, ATV-NE#3, ATV-NE#4, ATV-NE#5, and ATV-NE#6 at a concentration equivalent to 100 µg/mL ATV, in the absence or presence of PA. After incubation for 1 h, the cells were washed three times with ice-cold PBS (pH 7.4), then fixed with 4% formaldehyde and blocking buffer (0.3% Triton X-100 and 10% normal goat serum in PBS, pH 7.4). Next, they were incubated overnight with anti-human ASBT antibody (1:500) and stained with 10 µg/mL Alexa Fluor-546-labeled secondary antibody for 1 h. Finally, the nuclei were counterstained with DAPI for 5 min and the cells were mounted onto glass slides in mounting medium. Fluorescence images were examined by confocal laser scanning microscopy.

#### 2.6.2. Quantitative cellular uptake of ATV-NE by Caco-2 and ASBT-transfected MDCK cells

Quantitative evaluation of cellular ATV uptake was performed by treating Caco-2 cells cultured in six-well plates with ATV in water, ATV in 0.5% (v/v) dimethyl sulfoxide (DMSO), ATV-NE#1, ATV-NE#3, ATV-NE#4, ATV-NE#5, and ATV-NE#6 (all equivalent to 100 µg/mL ATV). Additionally, ATV infiltration from ATV in water, ATV in 0.5% (v/v) DMSO, ATV-NE#1, ATV-NE#4, ATV-NE#5, and ATV-NE#6 into ASBT-transfected or non-transfected MDCK cells was quantified in the absence or presence of an SMVT inhibitor.

After incubation for 1 or 3 h, the cell monolayers were washed three times with cold PBS (pH 7.4) and harvested after adding trypsin. After centrifugation, the cells were resuspended and washed with ice-cold PBS (pH 7.4). The collected cells were then treated with 1 mL of methanol to extract ATV. After centrifugation, 1 mL of each ATV-containing supernatant was mixed with 100 µL of a simvastatin solution (1,000 ng/mL) (the IS). The samples were dried on a rotary evaporator and reconstituted with the mobile phase (10 mM ammonium formate in 0.04% [v/v] formic acid:acetonitrile, 50:50, [v/v]). The ATV levels were then analyzed using a UPLC-MS/MS system as described below.

#### 2.6.3. UPLC–MS/MS analysis

Samples (10 µL) were injected into a UPLC system (ACQUITY UPLC; Waters Corp., Milford, MA, USA) equipped with an ACQUITY UPLC BEH C18 column (2.1 × 100 mm, 1.7 µm; Waters Corp.) at 40 °C. ATV and IS were eluted by the mobile phase at a flow rate of 0.4 mL/min with a time gradient of mobile phase B (acetonitrile): 30% (initial), 30% (1.0 min), 60% (1.5 min), 80% (2.0 min), 95% (2.5 min), 95% (3.0 min), 80% (3.5 min), 60% (4 min), 30% (5 min), and 30% (6 min) (all v/v). A mass spectrometer (Xevo TQ-S; Waters Corp.) was used for ionization of ATV or the IS (in positive ion multiple reaction-monitoring mode) at an electrospray voltage of 3.0 kV, with a source temperature of 150 °C, desolvation temperature of 500 °C, and desolvation gas flow rate of 800 L/h. ATV quantification was performed by reference to the m/z transition 559.2 → 440.2 at a cone voltage of 45 V and collision energy of 22 eV; for IS, the m/z transition was 436.3 → 285.2 at a cone voltage of 18 V and collision energy of 13 eV (El-Zailik et al., 2019). The lowest limit of quantification (LOQ) of ATV was 0.1 ng/mL. The calibration curve was linear over the concentration range 0.1–250 ng/mL for ATV; the linearity (r^2^) of ATV was 0.998 ± 0.002 with a coefficient of variation (CV) of 0.16%. The intra- and inter-day values were 3–6%.

### 2.7. Intestinal transport mechanism of ATV-NEs

To examine pathways involved in the transcellular permeation of ATV-NEs, a Caco-2/HT-29 MTX-E12 cell monolayer was prepared as described above. Then, 0.1 mL of HBSS containing specific transporter inhibitors and 0.6 mL of HBSS were added to the apical and basolateral chambers of each well, respectively (Table S1); this was followed by incubation for 30 min at 37 °C. After equilibration, the solution in the apical chamber was replaced with 0.1 mL of ATV-NEs diluted with HBSS (equivalent to 100 µg/mL ATV), along with a specific transport inhibitor (Table S1). Thereafter, the solution in the basolateral chamber was replaced with 0.6 mL of fresh HBSS and the cells were incubated at 37 °C. Then, 200-µL aliquots of the sample solution were withdrawn from the basolateral compartment at predetermined time points (0.5, 1, 2, 3, 4, and 5 h); this was followed by replacement with the same volume of fresh HBSS.

Separately, to examine the involvement of P-gp efflux in the permeation of ATV from ATV-NE#6, the *P_app_* value of ATV from apical to basal with or without Cys A (a specific P-gp efflux inhibitor) was evaluated. Furthermore, to explore the paracellular transport of ATV during the permeation of ATV-NE#6, a Caco-2 cell monolayer with a TEER value of ≥ 450 Ω·cm^2^ on the apical side was treated with 0.1 mL of Ca^2+^-free HBSS containing 2.5 mM EGTA (an intracellular tight junction opener) in the presence or absence of all inhibitors excluding Cys A. In contrast, the receptor compartment was filled with 0.6 mL of Ca^2+^-free HBSS. After preincubation at 37 °C for 45 min, the integrity of the monolayer was evaluated to determine whether the TEER value was maintained at ≤ 70 Ω·cm^2^; this confirmed the opening of junctional sites in the cell monolayer. Then, the solution on the apical side was replaced with 0.1 mL of HBSS containing 1.8 mM Ca^2+^ and ATV-NE#6 (equivalent to 100 µg/mL ATV) alone or with all inhibitors excluding Cys A. Subsequently, 0.6 mL of HBSS with 1.8 mM Ca^2+^ was added to each basolateral chamber, and the cells were incubated at 37 °C. Then, 200-µL aliquots of the sample solution were collected from the receptor compartment and replaced with the same volume of fresh HBSS containing 1.8 mM Ca^2+^. Additionally, the integrity of the cell monolayer was monitored by measuring the TEER value, which was ≥ 400 Ω·cm^2^ at 5 h after drug treatment; this indicated restoration of transmembrane resistance.

ATV that infiltrated across the cell monolayer was extracted with methanol and quantified by UPLC-MS/MS, as described in Section 2.6.3. Finally, *P_app_* values were estimated from the linear slopes of the cumulative amounts of ATV that permeated from the ATV-NEs over time, using the equation in Section 2.5.

### 2.8. In vitro cytotoxicity analysis

To evaluate the cytotoxic effects of ATV-NEs, 4T1 (murine stage IV breast cancer) or MCF-7 (human breast adenocarcinoma) cells were seeded in 96-well plates at a density of 1 × 10^4^ cells/well in 100 µL of Roswell Park Memorial Institute medium or Dulbecco’s Modified Eagle’s Medium supplemented with 10% FBS, 1% penicillin/streptomycin, and 1% non-essential amino acids. The cells were cultured for 24 h at 37 °C and starved for an additional 24 h in medium containing 1% FBS. The cells were then treated with ATV in water, ATV in 0.5% DMSO, ATV-NE#1, ATV-NE#4, ATV-NE#5, and ATV-NE#6, all of which were serially diluted with blank medium to ATV concentrations equivalent to 1, 5, 10, 20, 50, 100, and 200 µg/mL. After incubation for 24 h, the cells were treated with 10 µL of 2-(2-methoxy-4-nitrophenyl)-3-(4-nitrophenyl)-5-(2,4-disulfophenyl)-2H-tetrazolium monosodium salt (WST-8) solution. After incubation for 1 h, the absorbance at 450 nm was measured and the percentage of viable cells was calculated by comparison with the absorbance of untreated cells (100% viability).

### 2.9. In vitro cell adhesion, invasion, and migration assays

The inhibitory effects of ATV or ATV-NEs on 4T1 cell adhesion to the extracellular matrix (ECM) were examined using a 48-well ECM cell adhesion kit (Cell Biolabs CytoSelect™ Cell Adhesion Kit; Cell Biolabs, Inc., San Diego, CA, USA), in accordance with the manufacturer’s instructions. Briefly, 4T1 cells were seeded into wells precoated with five different proteins present in ECM (i.e., fibronectin, collagen II, collagen IV, laminin I, fibrinogen, and bovine serum albumin [negative control]) at a density of 1 × 10^6^ cells/well, along with drug solution at a concentration equivalent to 100 µg/mL ATV. After incubation for 1.5 h at 37 °C, the cells were washed with PBS (pH 7.4) and stained by incubation for 10 min with the dye included in the kit. Then, each well was gently washed five times with 500 µL of sterilized water; 200 µL of extraction solution were added to each well, and the cells were incubated for 10 min in an orbital shaker. Finally, change in color was measured with a plate reader at 560 nm; inhibition of adhesion in the drug-treated cells was evaluated relative to inhibition in the untreated cells (control).

The *in vitro* inhibition of 4T1 cell invasion and migration by ATV-NEs was assessed by Cultrex^®^ 24-well basement membrane extract cell invasion assays (Trevigen, Inc., Gaithersburg, MD, USA). Briefly, the membrane insert in each well was uniformly coated with 100 µL of 0.5× basement membrane extract and incubated for 24 h at 37 °C. Separately, uncoated wells were used for cell migration assays. Next, each coated well was washed using 1× buffer. For invasion assays, the cells in Roswell Park Memorial Institute medium with 0.5% FBS were seeded into each apical chamber at a density of 1 × 10^6^ cells/well. For cell migration assays, the same number of cells was seeded in the uncoated apical compartment. Subsequently, 500 µL of Roswell Park Memorial Institute medium containing 10% FBS were loaded into the basal compartment to serve as a chemoattractant; the cells were then cultured at 37 °C. After incubation for 24 h, both apical and basal compartments were washed with 1× buffer and the basal chamber was filled with 500 µL of cell dissociation solution (calcein-AM); the cells were incubated for 1 h at 37 °C. Finally, the dissolved calcein-AM concentration was measured with a fluorescence reader at an excitation wavelength of 485 nm and emission wavelength of 520 nm.

### 2.10. In vivo oral absorption in rats

To evaluate the enhanced oral absorption of ATV from the ATV-NEs after inclusion of DOCA-TAP and Biotinyl PE, Sprague–Dawley rats were orally administered 400 µL of ATV in water (40) (40 mg/kg ATV dispersed in water), ATV in 10% dimethylformamide (DMF) (40 mg/kg ATV dissolved in 10% DMF), or aqueous dispersion of ATV-NE#1 (40) (equivalent to 40 mg/kg ATV) or ATV-NE#6 (40) (equivalent to 40 mg/kg ATV). To calculate the relative oral bioavailability, rats were intraperitoneally (IP) injected with 200 µL of ATV-IP (2) (2 mg/kg ATV dissolved in 10% DMF). Then, 150-μL blood samples were collected from the femoral vein at predetermined time points into heparinized tubes and immediately centrifuged at 10,000 × *g* for 10 min at 4 °C. The separated plasma samples were kept frozen at −80 °C until analysis. To determine the plasma concentrations of ATV, 100 µL of each plasma sample was spiked with 100 µL of an IS solution (10 ng/mL simvastatin). Next, 1 mL of a mixture of acetonitrile and methanol (50:50, v/v) was added to each plasma sample spiked with IS, followed by vortex mixing and extraction. After centrifugation at 10,000 rpm for 5 min at 4 °C, the organic phase was transferred to another vial and evaporated to dryness on a rotary evaporator) at a reduced boiling point. Finally, the residues were reconstituted in 100-µL amounts of the mobile phase (10 mM ammonium formate in 0.04% [v/v] formic acid:acetonitrile, 50:50, [v/v]) and the concentration of ATV in each sample was determined by UPLC-MS/MS, as described in Section 2.6.3.

### 2.11. In vivo tumor growth inhibition of ATV-NEs

To evaluate the *in vivo* antitumor efficacy of oral ATV-NEs, 1 × 10^6^ 4T1 cells dispersed in 100 μL of PBS (pH 7.4) were subcutaneously inoculated into the left flanks of BALB/c mice. After the tumors reached 60–70 mm^3^ each, the mice were randomly divided into seven groups (*n* = 10 per group): control (untreated), ATV-IP (5) (once-daily IP administration of 5 mg/kg ATV in 10% DMF), ATV-IP (10) (once-daily IP administration of 10 mg/kg ATV in 10% DMF), ATV-oral (40) (once-daily oral administration of 40 mg/kg ATV in 10% DMF), ATV-NE#6 (10) (once-daily oral administration ATV-NE#6 dispersed in water, equivalent to 10 mg/kg ATV), ATV-NE#6 (20) (once-daily oral administration ATV-NE#6 dispersed in water, equivalent to 20 mg/kg ATV), and ATV-NE#6 (40) (once-daily oral administration ATV-NE#6 dispersed in water, equivalent to 40 mg/kg ATV). To assess tumor growth suppression, the tumor size and body weight of the mice in all groups were measured every 3 days after treatment. The tumor volume in each mouse was calculated as (width)^2^ × length × 0.52. After 21 days of treatment, mice were sacrificed and tumor masses were measured.

### 2.12. Pharmacokinetics and statistical analyses

Pharmacokinetic parameters were determined using a non-compartmental method in WinNonlin^®^ software (ver. 5.3; Pharsight Corporation, Mountain View, CA, USA). All values are expressed as means ± standard deviations (SDs) or standard errors of the mean (SEM). Statistical analyses for comparisons among ≥ 3 mean values for unpaired data were performed by one-way analysis of variance, followed by Tukey’s multiple comparisons test. In all analyses, *p* < .05 was considered indicative of statistical significance.

## 3. Results and discussion

### 3.1. Preparation and characterization of ATV-NEs

To enhance the aqueous solubility and permeability of ATV, o/w NEs were prepared by dispersing ATV in Capryol 90 as the oil phase, with S_mix_ (Tween 80:Transcutol HP, 1:2, w/w) as an emulsifier to maintain the stability of NEs in the aqueous phase (Table 1). Additionally, TPGS was incorporated in the S_mix_ to promote ATV solubility and permeation. These basic NEs of ATV (i.e., ATV-NE#1) had a particle size of 6.65 ± 0.27 nm with zeta potential of −4.68 ± 0.25 mV (Table 2). Furthermore, with the inclusion of DOTAP or DOCA in ATV-NE#2 and ATV-NE#3, the zeta potentials were maintained at +2.88 ± 0.85 mV and −8.50 ± 1.85 mV, respectively (Table 2). The positive zeta potential of ATV-NE#2 compared with the zeta potential of ATV-NE#1 may have been caused by the presence of the positive lipid DOTAP, whereas the zeta potential of ATV-NE#3 was higher than the zeta potential of ATV-NE#1; these findings suggested localization of the negative part of DOCA on the outer surface of the NEs. The decreased zeta potential of ATV-NE#4 may have been related to the neutralization of DOTAP by DOCA after ionic complex formation and the anchoring of DOCA-TAP on the surface of NE droplets. Similarly, ATV-NE#5 possessing Biotinyl PE showed a negative zeta potential, confirming its anchorage to the oil droplets with surfactant and co-surfactant.

**Table 2.** Particle sizes, polydispersity indices, zeta potentials, and aqueous solubilities of selected ATV-NEs.

Test material	Particle size (nm)	Polydispersity index	Zeta potential (mV)	Aqueous solubility (mg/mL)
ATV in water				0.98 ± 0.01
ATV in 0.5% DMSO				1.82 ± 0.01
ATV-NE#1	6.65 ± 0.27	0.22 ± 0.07	−4.68 ± 0.25	12.3 ± 0.14
ATV-NE#2	42.5 ± 5.82	0.60 ± 0.10	+2.88 ± 0.85	11.1 ± 0.07
ATV-NE#3	14.9 ± 0.23	0.21 ± 0.01	−8.50 ± 1.85	12.3 ± 0.27
ATV-NE#4	15.5 ± 0.42	0.24 ± 0.02	−1.39 ± 0.41	12.3 ± 0.07
ATV-NE#5	9.44 ± 0.04	0.19 ± 0.01	−5.08 ± 0.86	12.5 ± 0.07
ATV-NE#6	17.3 ± 0.50	0.27 ± 0.03	−3.78 ± 0.16	12.5 ± 0.15

All values are shown as means ± SDs (*n* = 3).

Furthermore, all ATV-NEs spontaneously formed o/w NEs with a homogeneous droplet size after dispersion into the aqueous phase (Table 2). ATV-NE#6 incorporating both DOCA-TAP and Biotinyl PE had a droplet size, polydispersity index, and zeta potential of 17.3 ± 0.50 nm, 0.27 ± 0.02, and −3.78 ± 0.16 mV, respectively (Figure 1(B), Table 2). Particle size analysis revealed that the morphology and surface structure of ATV-NE#6 were nanosized droplets < 50 nm in diameter, as measured by transmission electron microscopy (Figure 1(C)).

These findings confirmed that S_mix_ in NEs established thermodynamically stable nanodispersions that minimized the interfacial energy; moreover, DOCA and biotin were sufficiently exposed to the outer aqueous phase after co-anchoring of DOCA-TAP and Biotinyl PE to the oil.

### 3.2. In vitro permeability of ATV-NEs

**Figure 2.**
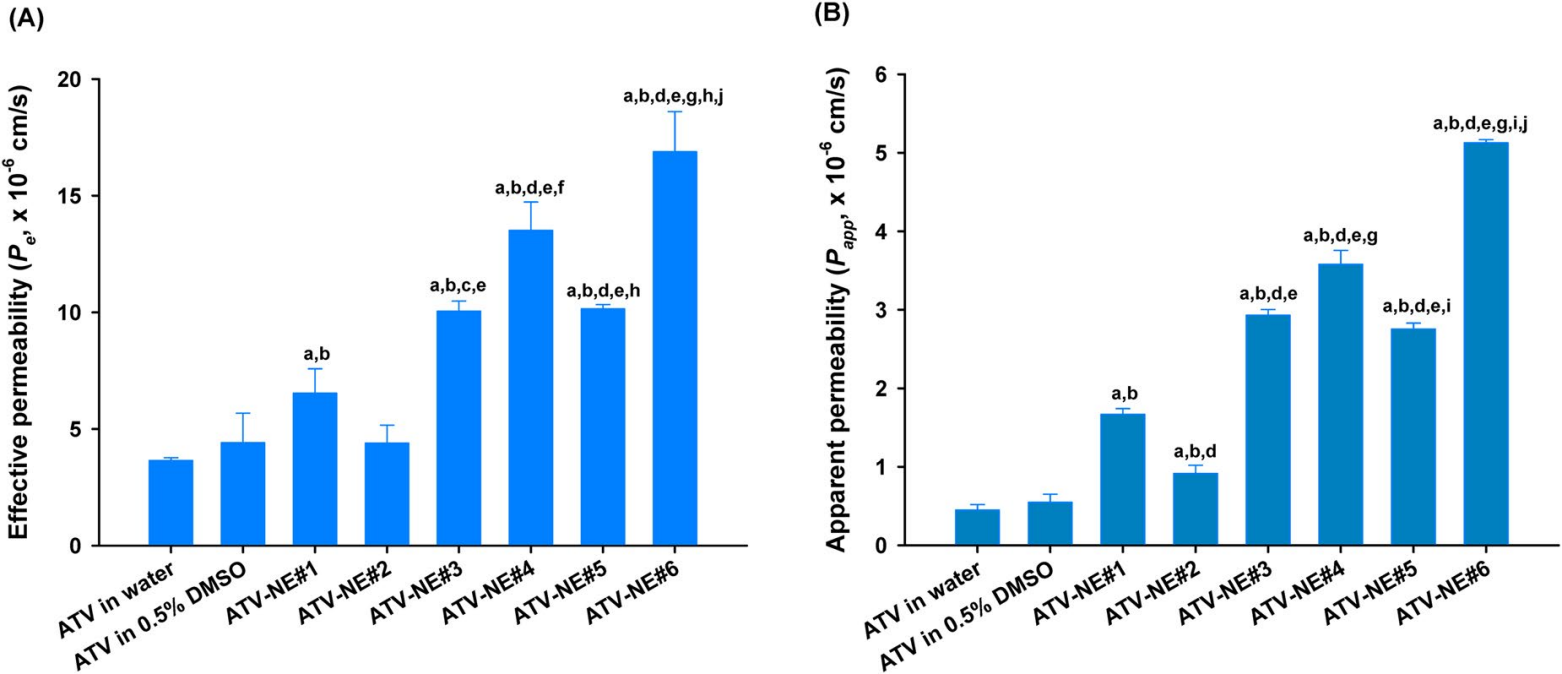
In vitro permeabilities of ATV-NEs. (A) Effective permeabilities (*P_e_*) of ATV and ATV-NE formulations through an artificial intestinal membrane. (B) Apparent permeability (*P_app_*) of ATV and ATV-NE formulations across a Caco-2/HT-29 MTX E12 cell monolayer. Values are shown as means ± SDs (*n* = 4 per group). ^a^*p* <.001 compared with ATV in water; ^b^*p* <.001 compared with ATV in 0.5% DMSO; ^c^*p* <.01, ^d^*p* <.001 compared with ATV-NE#1; ^e^*p* <.001 compared with ATV-NE#2; ^f^*p* <.01, ^g^*p* <.001 compared with ATV-NE#3; ^h^*p* <.01, ^i^*p* <.001 compared with ATV-NE#4; ^j^*p* <.001 compared with ATV-NE#5.

As shown in Figure 2(A), ATV in 0.5% DMSO enhanced the *P_e_* of ATV by 21.1%, compared with ATV in water. Furthermore, the *P_e_* of ATV-NE#1 was increased by 47.8% and 79.0%, compared with the respective *P_e_* values of ATV in water and ATV in 0.5% DMSO. This enhanced permeability may have been caused by the improved aqueous solubility of ATV after incorporation into ATV-NE#1. In contrast, the artificial membrane permeability of ATV from ATV-NE#2 was decreased compared with the permeability of ATV from ATV-NE#1, which may have been related to the greater hydrophilicity of DOTAP incorporating ATV-NE#2, rather than ATV-NE#1. However, after the inclusion of DOCA, DOCA-TAP, or Biotinyl PE alone into ATV-NE#1 (i.e., ATV-NE#3, ATV-NE#4, and ATV-NE#5, respectively), their *P_e_* values were increased by 53.8%, 107%, and 55.4%, compared with the *P_e_* value of ATV-NE#1. Furthermore, ATV-NE#6 exhibited 158% higher *P_e_*, compared with ATV-NE#1; this resulted in a 362% increase in the *P_e_* of ATV compared with ATV dispersed in water.

Next, the *P_app_* values of ATV from the various formulations across the Caco-2/HT-29 MTX E12 cell monolayers were examined (Figure 2(B)). The *P_app_* of ATV-NE#1 was increased by 204% and 272% compared with ATV in 0.5% DMSO and ATV in water, respectively; these findings suggested that S_mix_ and TPGS disrupt the epithelial barrier while inhibiting P-gp-mediated drug efflux. Similarly, the *P_app_* of ATV was increased by an additional 1.76-, 2.15-, and 1.65-fold after DOCA, DOCA-TAP, or Biotinyl PE had been anchored to ATV-NE#1 (i.e., ATV-NE#3, ATV-NE#4, and ATV-NE#5), respectively; these results indicated that DOCA in DOCA-TAP or biotin in Biotinyl PE may also promote ASBT or SMVT-mediated transport of ATV-NEs. Moreover, the *P_app_* of ATV-NE#6 was improved by an additional 43.3% and 86.1% compared with the *P_app_* values of ATV-NE#4 and ATV-NE#5; this resulted in values that were 207% and 1044% higher than the values of ATV-NE#1 and free ATV dispersed in water, respectively. Therefore, the permeability of ATV was significantly facilitated by the synergistic actions of the nanoemulsive system for enhancing drug solubility and membrane fluidity, as well as ASBT- and SMVT-mediated endocytosis via DOCA- and biotin-conjugated lipids.

### 3.3. Cellular uptake of ATV-NEs in Caco-2 and ASBT-transfected MDCK cells

Next, we analyzed the intracellular uptake of various ATV-loaded nanoemulsive systems into Caco-2 cells. As shown in Figure 3(A), the results of in vitro permeability studies were consistent with the results of confocal microscopy. ATV-NE#1 containing coumarin 6 showed enhanced uptake compared with free ATV in water or in 0.5% DMSO. This enhanced uptake was directly related to the increases in drug solubility and permeability after incorporation into NEs consisting of Tween 80, Transcutol HP, and TPGS, which have been shown to change membrane structure (e.g., fluidity, endocytosis, and macropinocytosis) and possibly suppress P-gp-mediated efflux (Plaza-Oliver et al., 2021; Collnot et al., 2010). After the inclusion of DOCA, DOCA-TAP, or Biotinyl PE, ATV-NE#3, ATV-NE#4, and ATV-NE#5 showed greater improvement of cellular uptake of coumarin 6 compared with ATV-NE#1, indicating that ASBT- or SMVT-mediated uptake during transport (Figure 3(A)).

Figure 3.In vitro cellular uptake of ATV-NEs. (A) Confocal laser scanning micrographs of cellular uptake of coumarin 6-coloaded ATV in water, ATV in 0.5% DMSO, ATV-NE#1, ATV-NE#3, ATV-NE#4, ATV-NE#5, and ATV-NE#6 by Caco-2 cells. Scale bar, 20 µm. (B) Quantitative analyses of cellular uptake of ATV in water, ATV in 0.5% DMSO, ATV-NE#1, ATV-NE#3, ATV-NE#4, ATV-NE#5, and ATV-NE#6 by Caco-2 cells after 1 h and 3 h of treatment. Values are shown as means ± SDs (*n* = 4 per group). ^a^*p* <.05, ^b^*p* <.001 compared with ATV in water; ^c^*p* <.001 compared with ATV in 0.5% DMSO; ^d^*p* <.001 compared with ATV-NE#1; ^e^*p* <.001 compared with ATV-NE#3; ^f^*p* <.001 compared with ATV-NE#4; ^g^*p* <.001 compared with ATV-NE#5. Confocal laser scanning micrographs of cellular uptake of coumarin 6-coloaded ATV in water, ATV in 0.5% DMSO, ATV-NE#1, ATV-NE#4, ATV-NE#5, and ATV-NE#6 by (C) MDCK (ASBT-non-transfected) or (D) ASBT-expressing MDCK cells with SMVT inhibitor, and (E) MDCK (ASBT-non-transfected) or (F) ASBT-expressing MDCK cells without SMVT inhibitor. Scale bar, 20 µm. Quantitative analyses of cellular uptake of ATV in water, ATV in 0.5% DMSO, ATV-NE#1, ATV-NE#4, ATV-NE#5, and ATV-NE#6 by MDCK (non-transfected) or ASBT-expressing MDCK cells with or without SMVT inhibitor after (G) 1 h and (H) 3 h of treatment. Values are shown as means ± SDs (*n* = 4 per group). **p <.01, ***p <.001 compared with uptake in ASBT-non-transfected MDCK cells treated with SMVT inhibitor; ###*p* <.001 compared with uptake in ASBT-expressing MDCK cells treated with SMVT inhibitor; $*p* <.05, $$$*p* <.001 compared with uptake in ASBT-non-transfected MDCK cells without SMVT inhibitor.

**Figure:**
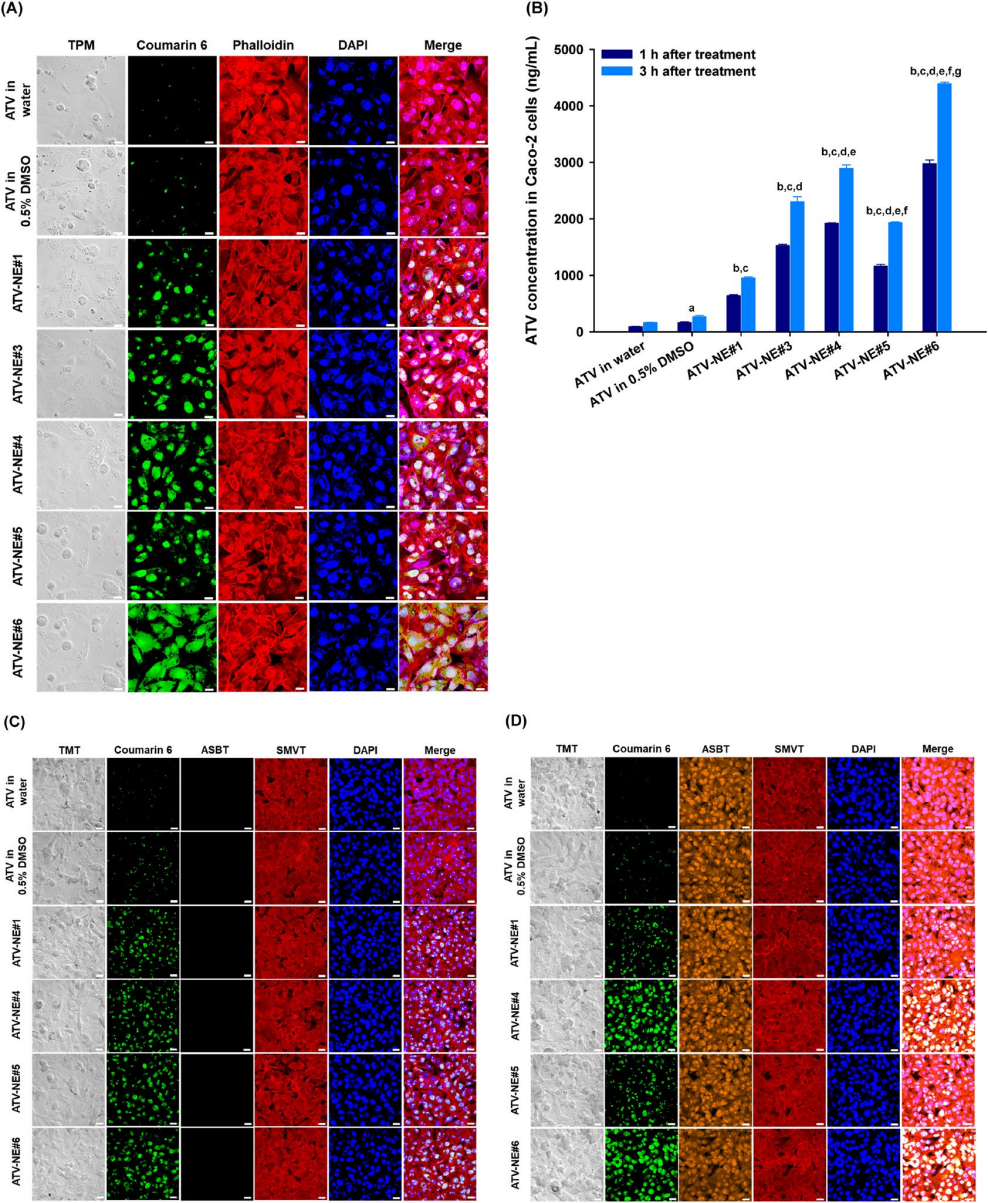

**Figure:**
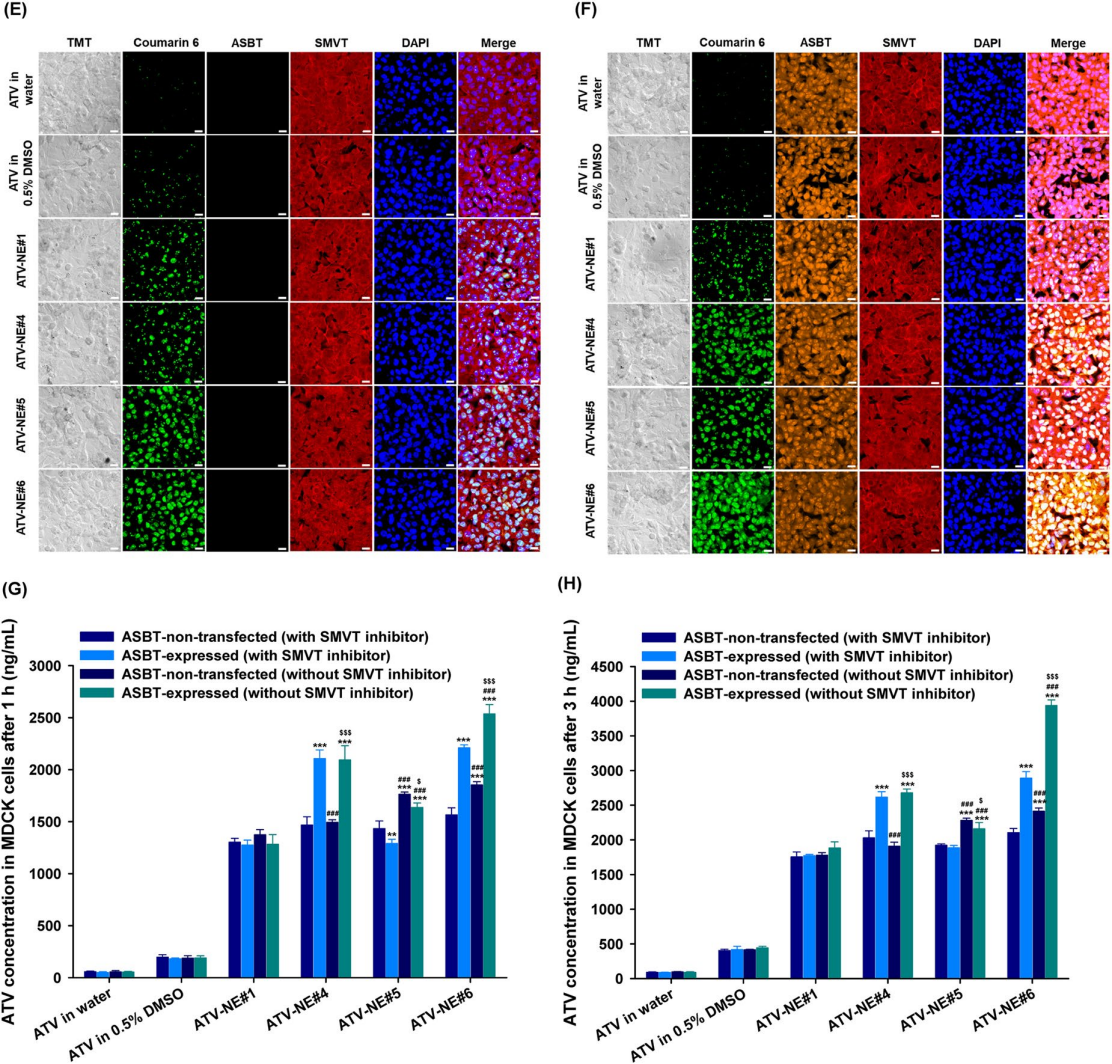

Furthermore, after both DOCA-TAP and Biotinyl PE had been physically anchored onto the ATV-NE#6, the cellular uptake of coumarin 6 was markedly increased compared with ATV-NE#4 and ATV-NE#5. These results were closely correlated with the intracellular concentration of ATV in Caco-2 cells at 1 or 3 h after treatment (Figure 3(B)). Thus, at 3 h after treatment with ATV-NE#6, the ATV levels in the cells were 4.62-, 1.91-, 1.52-, and 2.27-fold greater than the levels in cells treated with ATV-NE#1, ATV-NE#3, ATV-NE#4, and ATV-NE#5, respectively; these results confirmed the synergistic actions of DOCA-TAP and Biotinyl PE during the permeation of ATV-NE#6 via ASBT- and SMVT-mediated transport.

Next, to confirm the dual targeting of DOCA- and biotin-linked lipids to ASBT and SMVT during infiltration, ASBT-expressing or non-transfected MDCK cells were exposed to coumarin 6-containing ATV-NEs in the presence or absence of SMVT inhibitor (i.e., PA). After treatment with free ATV in water or 0.5% DMSO, as well as ATV-NE#1, confocal imaging did not reveal any variation in cellular uptake, regardless of the presence or absence of ASBT and/or SMVT inhibitor. However, ATV-NE#4 showed greater absorption into cells expressing ASBT, regardless of the presence of SMVT inhibitor. Furthermore, ATV-NE#5 showed enhanced uptake of coumarin 6 in PA-untreated MDCK cells. For ATV-NE#6, the uptake of coumarin 6 was greater in ASBT-expressing MDCK cells than in non-transfected MDCK cells and/or SMVT inhibitor-untreated cells (Figure 3(C–F)).

These results were consistent with the intracellular infiltration of ATV into ASBT-expressing or non-transfected MDCK cells, with or without SMVT inhibitor. After exposure to ATV-NE#4 for 1 h, the cellular uptake of ATV was 1.44-fold greater in ASBT-expressing MDCK cells (with SMVT inhibitor) than in ASBT-non-transfected MDCK cells (with SMVT inhibitor). At 1 h after treatment with ATV-NE#5, the cellular uptake of ATV was 1.20-fold greater in the ASBT-non-transfected cells (without SMVT inhibitor) than in ASBT-non-transfected cells (with SMVT inhibitor) or ASBT-expressing cells (with SMVT inhibitor) (Figure 3(G)). Consistent with these results, the infiltration of ATV from ATV-NE#6 at 3 h was significantly increased in cells expressing ASBT in the absence of SMVT inhibitor; this level was 1.87- and 1.63-fold greater than the levels in ASBT-non-transfected MDCK cells with or without SMVT inhibitor, respectively (Figure 3(H)). These findings confirmed the synergistic actions of DOCA-TAP and Biotinyl PE on ASBT- and SMVT-facilitated cellular uptake of ATV-NE#6.

### 3.4. Intestinal transport mechanism of ATV-NEs

**Figure 4.**
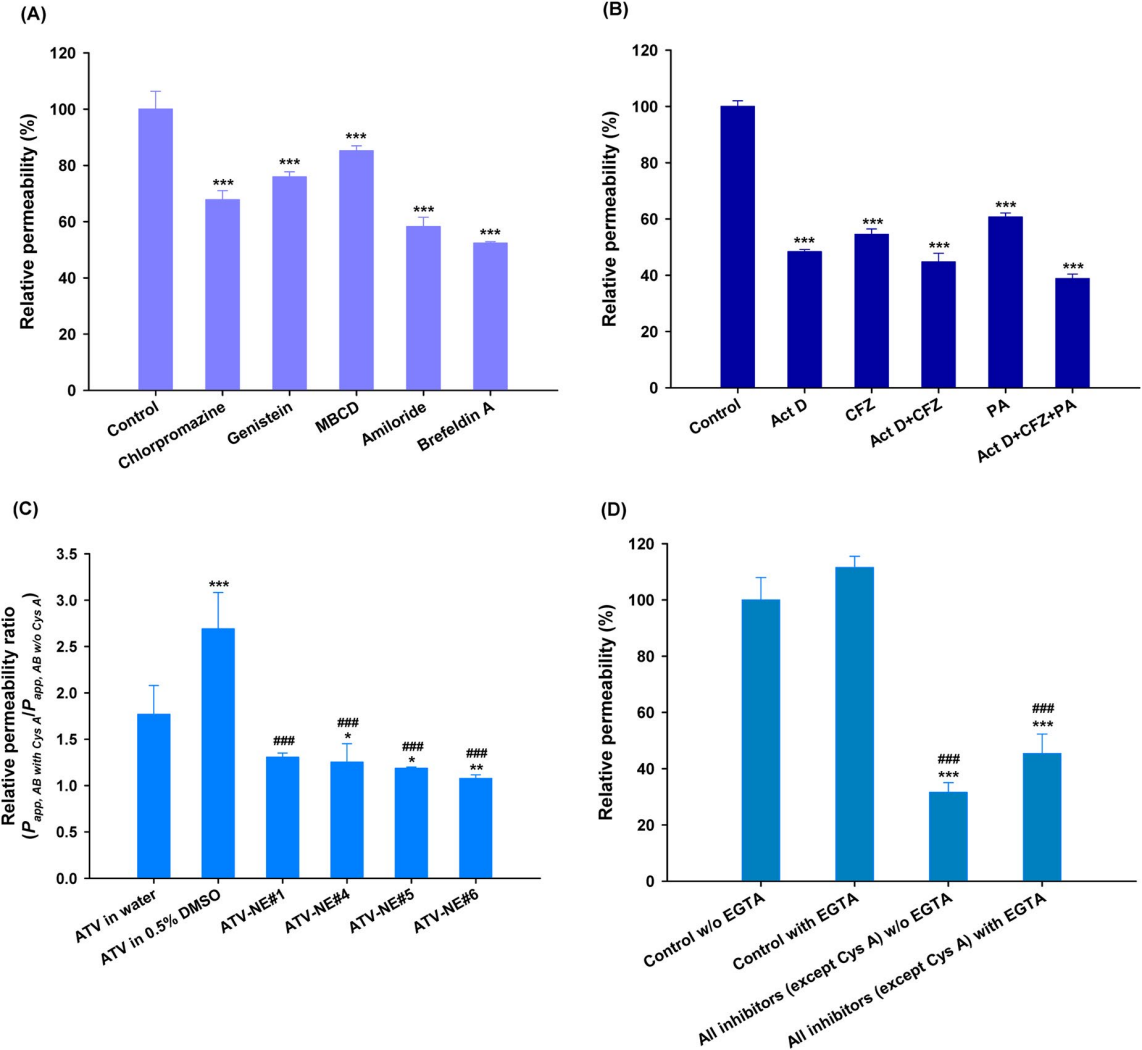
In vitro transport mechanism of ATV-NEs across Caco-2/HT-29 MTX E12 cell monolayers. (A) Relative apparent permeability (*P_app_*) values of ATV-NE#6 after incubation with various transport inhibitors of transcytosis mechanism. Values are shown as means ± SDs (*n* = 4). ^***^p <.001 compared with *P_app_* of ATV-NE#6 in the absence of all inhibitors (untreated control). (B) Relative *P_app_* values of ATV-NE#6 in the presence of inhibitors of ASBT, OST_α/β_, and SMVT alone or in various combinations. Values are shown as means ± SDs (*n* = 4). ^***^p <.001 compared with *P_app_* of ATV-NE#6 in the absence of all inhibitors (untreated control). (C) Relative *P_app_* ratios of ATV in water, ATV in 0.5% DMSO, ATV-NE#1, ATV-NE#4, ATV-NE#5, and ATV-NE#6 in the apical-to-basolateral (AB) direction in the presence or absence of Cys A (net *P_app_* ratio [NAPR]; *P_app, AB, with Cys A_*/*P_app, AB, w/o Cys A_*). Values are shown as means ± SDs (*n* = 4). ^*^p <.05, ^**^p <.01, ^***^p <.001 compared with NAPR value of ATV in water; ^###^*p* <.001 compared with NAPR value of ATV in 0.5% DMSO. (D) Relative *P_app_* values of ATV-NE#6 with or without EGTA in the presence or absence of all inhibitors other than Cys A. Values are shown as means ± SDs (*n* = 4). ^***^p <.001 compared with *P_app_* of ATV-NE#6 without EGTA in the absence of all inhibitors (untreated control); ^###^*p* <.001 compared with *P_app_* of ATV-NE#6 with EGTA in the absence of all inhibitors.

To confirm the involvement of specific pathways responsible for the increase in permeation of ATV from ATV-NEs, *P_app_* was evaluated after treatment with specific biochemical inhibitors for intestinal membrane transport. After treatment with chlorpromazine, the *P_app_* of ATV-NE#6 was decreased by 32.2% compared with the control (untreated), indicating that the clathrin-mediated pathway had an important role in the transport of ATV-NE#6 (Figure 4(A)). Additionally, genistein and MBCD reduced the *P_app_* of ATV-NE#6 by 24.1% and 14.8%, respectively, compared with the control (untreated); these findings indicated that caveola/lipid raft-mediated endocytosis was involved in the permeation of ATV-NE#6 (Figure 4(A)). An inhibitor of macropinocytosis, amiloride, reduced the *P_app_* of ATV-NE#6 by 41.8% compared with the control (untreated) (Figure 4(A)). To confirm the involvement of the ER/Golgi pathway during endosomal trafficking of ATV-NE#6, brefeldin A was used as a strong inhibitor; this treatment led to a 47.6% decrease in the *P_app_* of ATV-NE#6 compared with the control (untreated) (Figure 4(A)). Taken together, these results suggest that ATV-NE#6 can be internalized by endocytosis and macropinocytosis, followed by endosomal trafficking of ATV-NE#6 across the enterocyte membrane through the ER/Golgi pathway.

Next, to examine the role of ASBT-mediated transport in the permeation of ATV-NE#6, ASBT was inhibited using a prominent inhibitor, Act D; this caused a 51.6% decrease in the permeation of ATV-NE#6 (Figure 4(B)). The involvement of heteromeric organic solute transporter (OST) α and β (OST_α/β_) during penetration across the basolateral membrane of enterocytes was also investigated by using the specific inhibitor, CFZ, to block this transport. The *P_app_* of ATV-NE#6 was decreased by 45.5% and 55.3% compared with the control (untreated) after treatment with CFZ alone or in combination with Act D, respectively; these results suggested that ATV-NE#6 can interact with ASBT during entry into the cell, travel by ileal bile acid-binding protein-directed intracellular trafficking, and exit across the basolateral membrane of enterocytes via OST_α/β_. Additionally, the active involvement of SMVT-facilitated cellular uptake of ATV-NE#6 was examined by inhibition of this pathway using PA. As expected, the *P_app_* of ATV-NE#6 was decreased by 39.3% compared with the control (untreated) (Figure 4(B)). Furthermore, because both ASBT and SMVT were inhibited by combined treatment with Act D, CFZ, and PA, the *P_app_* of ATV-NE#6 was also significantly decreased by 61.2% compared with the control (untreated) (Figure 4(B)). Taken together, these results showed that DOCA (head) complexed with cationic lipid, DOTAP (tail), and biotin (head)-conjugated polar lipid (tail) can interact simultaneously with ASBT and SMVT, thus mimicking their functional ligands to facilitate absorption via ASBT- and SMVT-mediated transport.

In addition to the absorption mechanism, the effects of P-gp-mediated efflux on the intestinal permeation of ATV or ATV-NEs were examined by comparison of the net increase in the *P_app_* of ATV in the apical-to-basolateral direction (*P_app, AB_*), with or without Cys A (a well-known P-gp inhibitor), in terms of the net apparent permeability ratio (NAPR) (i.e., *P_app, AB, with Cys A_/P_app, AB, w/o Cys A_*). The presence of P-gp-mediated efflux was considered substantial if the NAPR (*P_app, AB with Cys A_ ≫ P_app, w/o Cys A_*) was >1. The NAPR values for ATV in water or 0.5% DMSO were 1.77 and 2.69, respectively, indicating that ATV is a substrate of P-gp and was effluxed during permeation (Figure 4(C)). In contrast, the *P_app, AB_* values of ATV-NE#1, ATV-NE#4, ATV-NE#5, and ATV-NE#6 did not substantially change regardless of the presence or absence of Cys A, with NAPR values of 1.1–1.3 (Figure 4(C)). These observations suggested that P-gp-mediated drug efflux also interfered with its oral absorption, which could be overcome by incorporating the drug into the nanoemulsive system composed of TPGS (i.e., an effective inhibitor of P-gp-mediated transport) (Kirtane et al., 2013; Sosnik, 2013).

Next, the involvement of paracellular transport was explored by determining the effects of EGTA on the *P_app_* of ATV-NE#6. EGTA is a selective calcium-chelating agent that can reversibly open the intracellular tight junctions of Caco-2 cell monolayers by binding with free extracellular Ca^2+^ ions.(Chuang et al., 2013) As shown in Figure 4(D), the *P_app_* values of ATV-NE#6 with or without all inhibitors other than Cys A did not significantly increase in the presence of EGTA, suggesting that the paracellular route plays a minimal role in its transport. Additionally, when monolayers were treated with all inhibitors other than Cys A (without EGTA), the *P_app_* of ATV-NE#6 was reduced by 68.4% compared with the control (without inhibitors and EGTA) (Figure 4(D)). Therefore, 31.6% of ATV-NE#6 may permeate mainly through transcellular pathways.

### 3.5. In vitro cytotoxicity studies

**Figure 5.**
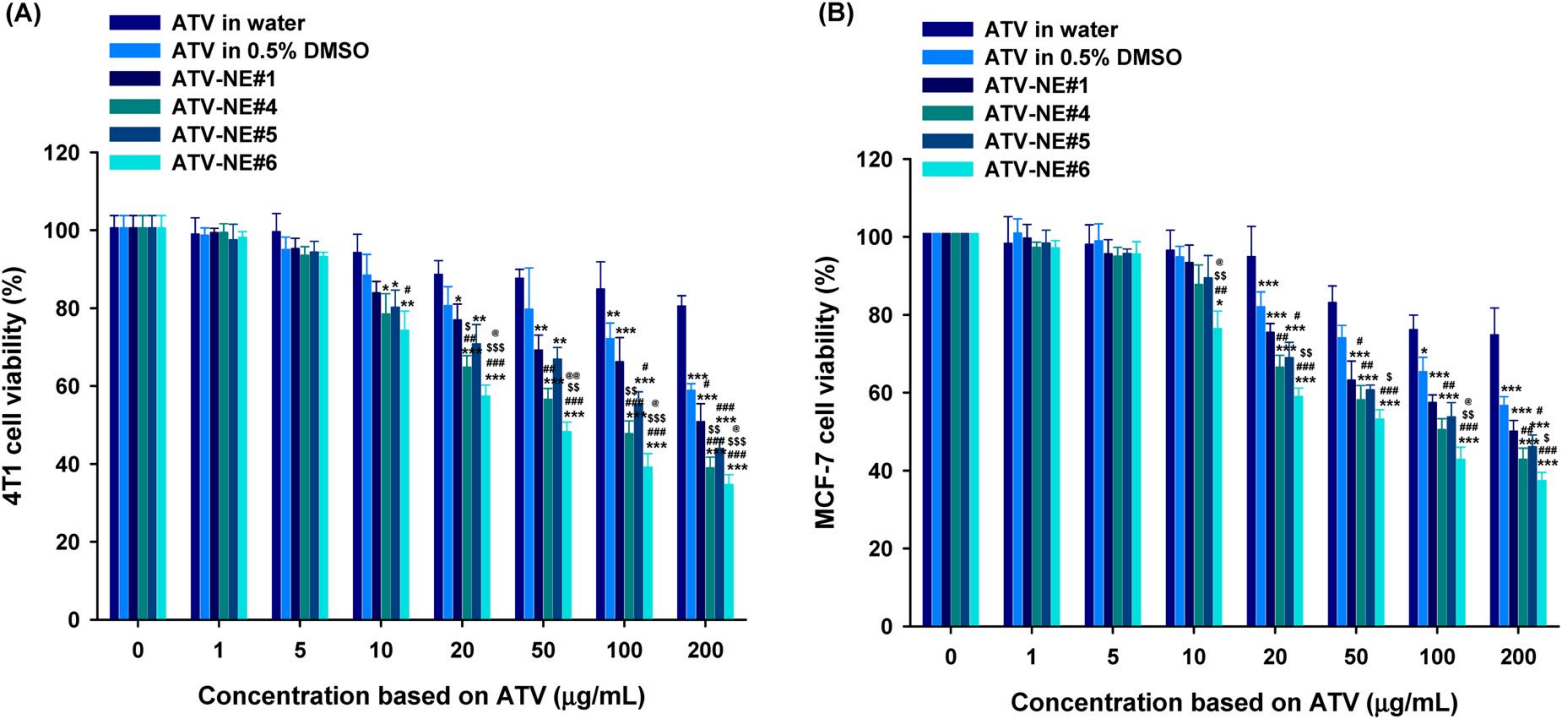
In vitro cytotoxic effects of ATV and ATV-NEs on (A) 4T1 and (B) MCF-7 cells after incubation for 24 h. Values are shown as means ± SDs (*n* = 4). ^*^p <.05, ^**^p <.01, ^***^p <.001 compared with ATV in water at a concentration equivalent to the concentration of ATV; ^#^*p* <.05, ^##^*p* <.01, ^###^*p* <.001 compared with ATV in 0.5% DMSO at a concentration equivalent to the concentration of ATV; ^$^*p* <.05, ^$$^*p* <.01, ^$$$^*p* <.001 compared with ATV-NE#1 at a concentration equivalent to the concentration of ATV; ^@^*p* <.05, ^@@^*p* <.01 compared with ATV-NE#5 at a concentration equivalent to the concentration of ATV.

The *in vitro* therapeutic potential of ATV formulations for inhibiting cancer cell proliferation was assessed by determining the cytotoxicity against 4T1 and MCF-7 cells (Figure 5). 4T1 and MCF-7 cells incubated with ATV dispersed in water (equivalent to 200 µg/mL ATV) showed viabilities of 80.5% ± 2.63% and 74.8% ± 6.91%, respectively, indicating that the low solubility and permeability of ATV limited its anticancer effect (Figure 5). In contrast, ATV in 0.5% DMSO exhibited cytotoxic effects toward 4T1 and MCF-7 cells at concentrations > 100 µg/mL ATV. Additionally, all ATV-loaded NEs exerted dose-dependent cytotoxic effects on MCF-7 and 4T1 cells at > 20 mg/mL of ATV (Figure 5). Furthermore, ATV-NEs showed significantly greater cytotoxic effects compared with ATV in 0.5% DMSO. After treatment for 24 h, the IC_50_ values for ATV-NE#1 and ATV-NE#6 in 4T1 cells were 198 ± 5.06 and 48.6 ± 2.30 µg/mL, respectively; these values were 1.29- and 5.27-fold lower than the IC_50_ value of ATV in 0.5% DMSO (256 ± 2.03 µg/mL). Similarly, the IC_50_ values for ATV-NE#1 and ATV-NE#6 in MCF-7 cells were 200 ± 4.87 and 58.3 ± 2.82 µg/mL, respectively, indicating 24.0% and 76.5% increases in the cytotoxic effects of ATV-NE#1 and ATV-NE#6 compared with ATV in 0.5% DMSO. In addition to the improvement of aqueous solubility, the further enhancement of cytotoxicity by ATV-NEs compared with ATV in 0.5% DMSO can be explained by enhanced cellular uptake along with the inhibition of multidrug resistance proteins (e.g. breast cancer receptor protein) expressed in MCF-7 and 4T1 cells by TPGS incorporated into the NEs (Jha et al., 2020a).

Overall, the results indicate that ATV anticancer activity is increased when the drug is formulated into a nanoemulsive system that enhances both drug solubility and membrane infiltration, in turn increasing tumor growth inhibition (via apoptosis and cell-cycle arrest) over the same dose range as that of the control cholesterol levels.

### 3.6. In vitro cell adhesion, invasion, and migration assays

**Figure 6.**
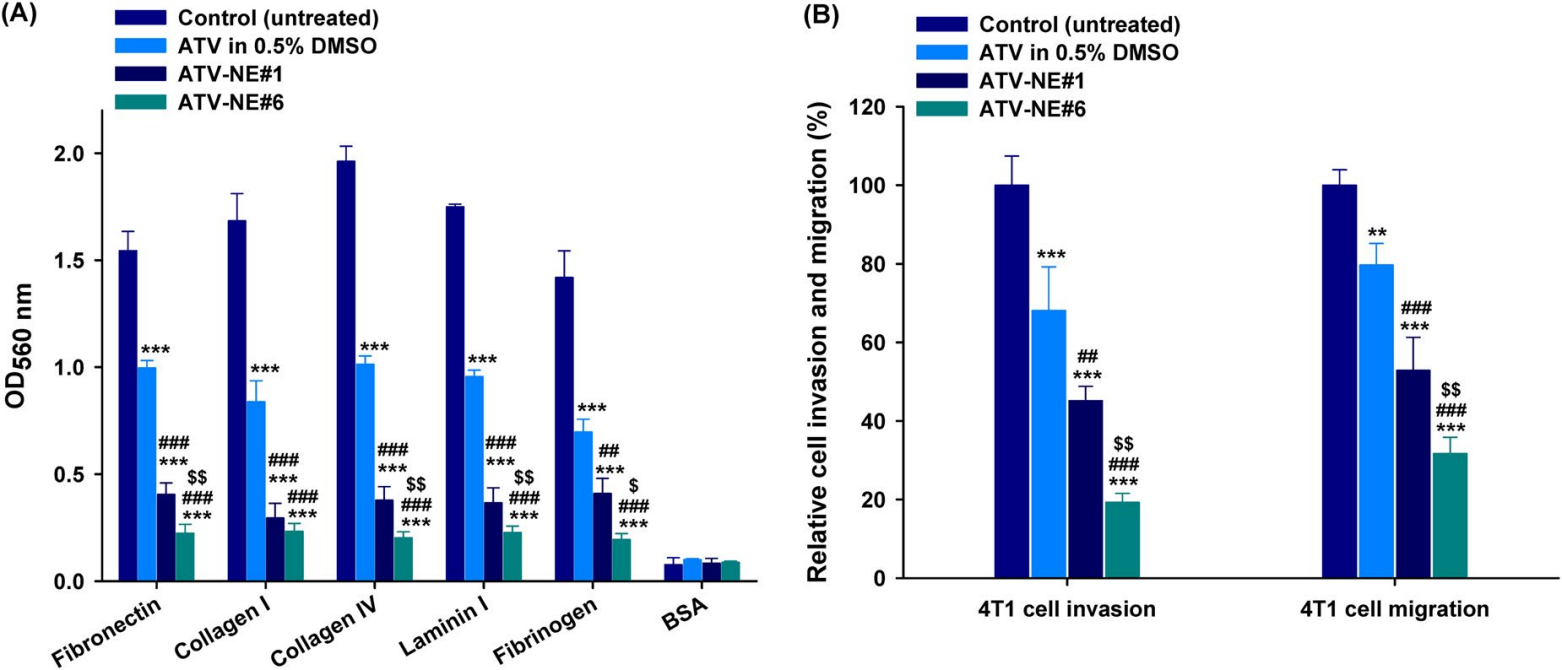
In vitro antimetastatic effects of ATV and ATV-NEs. (A) Inhibition of the adhesion of 4T1 cells to the extracellular matrix (ECM) components fibronectin, collagen I, collagen IV, laminin I, and fibrinogen, and bovine serum albumin (control) by ATV in 0.5% DMSO, ATV-NE#1, and ATV-NE#6. Values are shown as means ± SDs (*n* = 4). ^***^p <.001 compared with the control (untreated) for the same ECM component; ^##^*p* <.01, ^###^*p* <.001 compared with ATV in 0.5% DMSO for the same ECM component; ^$^*p* <.05, ^$$^*p* <.01 compared with ATV-NE#1 for the same ECM component. (B) Inhibition of invasion and migration of 4T1 cells by ATV in 0.5% DMSO, ATV-NE#1, and ATV-NE#6. Values are shown as means ± SDs (*n* = 4). ^**^p <.01, ^***^p <.001 compared with the control (untreated); ^##^*p* <.01, ^###^*p* <.001 compared with ATV in 0.5% DMSO; ^$$^*p* <.01 compared with ATV-NE#1.

Next, the antimetastatic activities of ATV in 0.5% DMSO, ATV-NE#1, and ATV-NE#6 to the 4T1 cells were assessed by *in vitro* cell adhesion, migration, and invasion assays. After incubation with ATV in 0.5% DMSO (100 µg/mL), 4T1 cells showed significantly decreased adhesion to ECM components (fibronectin, collagen I, collagen IV, laminin I, and fibrinogen) compared with the untreated control (Figure 6(A)). Furthermore, ATV-NE#1 and ATV-NE#6 suppressed 4T1 cell adhesion to the ECM components to a greater extent than ATV in 0.5% DMSO; compared with than ATV in 0.5% DMSO, ATV-NE#6 treatment resulted in 77.5%, 72.3%, 80.0%, 76.2%, and 72.0% greater inhibitory effects on cell adhesion to the fibronectin, collagen I, collagen IV, laminin I, and fibrinogen, respectively (Figure 6(A)).

Furthermore, treatment with ATV in 0.5% DMSO (100 µg/mL) impeded the invasion and migration of 4T1 cells by 31.9% and 20.3%, respectively, compared with untreated controls (Figure 6(B)). Similarly, cell invasion was considerably decreased by 54.8% and 80.7%, whereas cell migration was inhibited by 47.1% and 68.3%, compared with the control (untreated) after incubation for 24 h with ATV-NE#1 and ATV-NE#6 equivalent to 100 µg/mL ATV, respectively (Figure 6(B)). Consistent with the cytotoxic activity, the ATV-incorporated NEs showed greater inhibitory effects on cell invasion and migration, compared with free ATV in 0.5% DMSO, due to improvements in drug solubility and permeability. In particular, ATV-NE#6 treatment showed 71.7% and 57.3% greater decreases in cell migration, as well as 60.2% and 66.6% lower invasion capacity, compared with treatment with ATV in 0.5% DMSO and ATV-NE#1, respectively (Figure 6(B)).

These results imply that ATV strongly suppresses the migration and invasion of breast cancer cells, which may be attributable to the inhibition of geranylgeranylation of the Rho family of proteins (such as Rho A and Rho C), which are overexpressed in breast cancer (Kusama et al., 2006). In addition, the ability of ATV to inhibit cancer cell adhesion and invasion was further enhanced after incorporation into the NE formulation; this increased both ATV solubility and cancer cell infiltration by impeding P-gp-mediated efflux, even in the presence of serum.

Together, the observations suggest that oral delivery of ATV-NE#6 suppresses tumor cell growth and metastasis more effectively than conventional oral dosage forms.

### 3.7. In vivo oral absorption of ATV-NEs in rats

**Figure 7.**
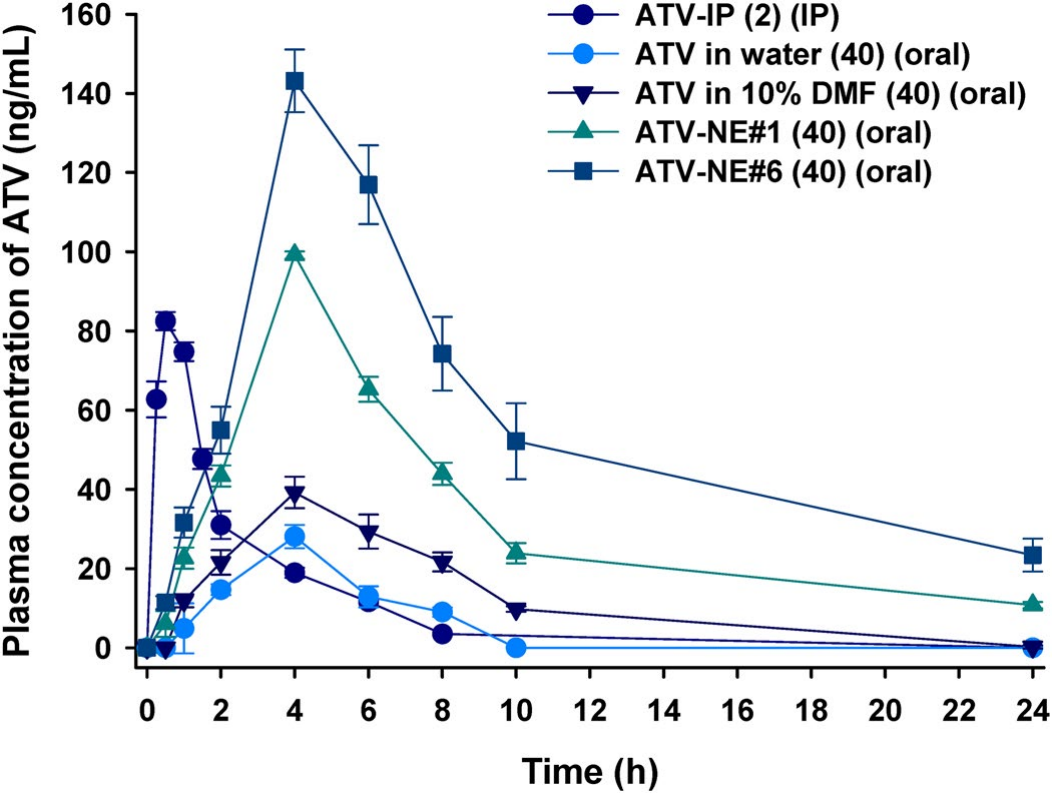
Venous plasma concentration–time profiles of ATV in rats after a single intraperitoneal (IP) injection of 2 mg/kg ATV [ATV-IP (2)] and oral administration of aqueous dispersion of 40 mg/kg ATV [ATV in water (40)]; 40 mg/kg ATV dissolved in 10% DMF [ATV in 10% DMF (40)]; aqueous dispersion of ATV-NE#1 as 40 mg/kg of ATV [ATV-NE#1 (40)]; or aqueous dispersion of ATV-NE#6 as 40 mg/kg of ATV [ATV-NE#6 (40)]. Values are shown as means ± SDs (*n* = 4).

Figure 7 and Table 3 present the plasma concentration–time profiles after IP or oral administration of ATV in the form of ATV in water, ATV in 10% DMF, ATV-NE#1, and ATV-NE#6 in Sprague–Dawley rats, along with the corresponding pharmacokinetic parameters. Compared with free ATV dispersed in water, the oral absorption of ATV solution in 10% DMF did not significantly improve. However, the plasma drug level was considerably increased after incorporation into normal NEs (i.e. ATV-NE#1). Compared with free ATV dispersed in water, the maximum plasma concentration (C_max_) and area under the plasma concentration–time (0–24 h) (AUC_last_) estimated after oral administration of ATV-NE#1 (40) were improved by 3.53- and 6.56-fold, compared with those parameters for ATV in water (40); they were also improved by 2.53- and 3.00-fold, compared with those parameters for ATV in 10% DMF (40). These improvements increased the relative oral bioavailability by 555% and 200% compared with ATV in water (40) and ATV in 10% DMF (40), respectively. The enhancement in oral absorption of ATV-NE#1 was further increased by physically anchoring DOCA-TAP and Biotinyl PE to the oil droplets in combination with S_mix_ (i.e., ATV-NE#6), which led to 42.3% and 81.8% greater increases in C_max_ and AUC_last_ of the ATV-NE#6 (40), compared with ATV-NE#1 (40). Thus, the relative oral bioavailability of ATV-NE#6 (40) was 33.1% ± 1.02%, which was 1091% and 446% greater than the corresponding values of ATV in water (40) and in 10% DMF (40), respectively.

**Table 3.** Pharmacokinetic parameters of ATV in rats after intraperitoneal (IP) or oral administration of ATV or ATV-NEs.

Test material	ATV-IP (2)	ATV in water (40)	ATV in 10% DMF (40)	ATV-NE#1 (40)	ATV-NE#6 (40)
Administration route	IP	Oral	Oral	Oral	Oral
ATV dose (mg/kg)	2	40	40	40	40
T_max_ (h)	0.50 ± 0.00	4.00 ± 0.00	4.00 ± 0.00	4.00 ± 0.00	4.00 ± 0.00
T_1/2_ (h)	1.77 ± 0.04	2.45 ± 0.26	2.97 ± 0.53	6.75 ± 0.33	9.72 ± 2.68
C_max_ (ng/mL)	82.5 ± 2.28	28.1 ± 2.94	39.2 ± 3.95	99.1 ± 0.92	141 ± 8.79
AUC_last_ (ng·h/mL)	211 ± 4.84	117 ± 7.03	256 ± 49.9	768 ± 34.7	1396 ± 43.1
AUC_inf_ (ng·h/mL)	220 ± 4.94	149 ± 13.5	283 ± 32.5	873 ± 29.4	1751 ± 170
Relative bioavailability (%)	100	2.78 ± 0.17	6.06 ± 1.18	18.2 ± 0.82	33.1 ± 1.02

Relative bioavailability (%) = (AUC_last, oral_/Dose_ATV, oral_)/(AUC_last, IP_/Dose_ATV, IP_) × 100. All values are shown as means ± SDs (*n* = 4). T_max_, time to reach maximum plasma concentration of ATV; T_1/2_, plasma half-life of ATV; C_max_, maximum plasma ATV concentration; AUC_last_, area under the plasma concentration–time curve between zero and the last measurable plasma concentration; AUC_inf_, area under the plasma concentration–time curve between zero and infinity.

The enhancement of the oral bioavailability of ATV-NE#6 was consistent with the results of *in vitro* permeability and mechanistic analyses. Possible explanations for the improved oral bioavailability include multimodal transport mechanisms, endocytosis and macropinocytosis of nanoemulsive systems, ASBT- and SMVT-facilitated transport by DOCA-TAP and Biotinyl PE, and suppression of P-gp-mediated efflux by TPGS in ATV-NE#6.

### 3.8. In vivo tumor growth inhibition by ATV-NE in 4T1-cell-xenografted mice

**Figure 8.**
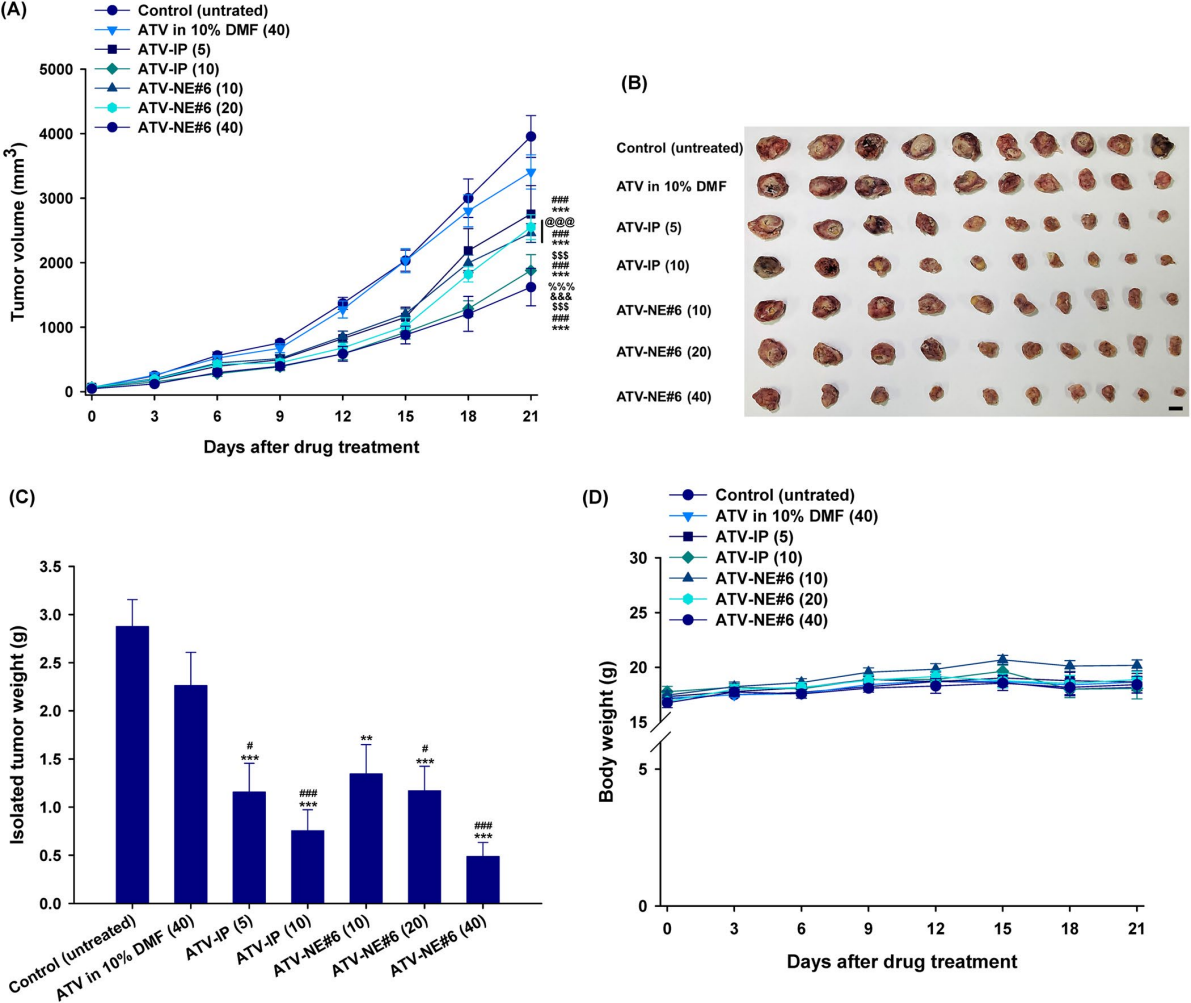
In vivo tumor growth inhibition in 4T1-cell-bearing mice after once-daily intraperitoneal (IP) administration of 5 and 10 mg/kg of ATV in 10% DMF [ATV-IP (5) and ATV-IP (10), respectively] and once-daily oral administration of 40 mg/kg of ATV in 10% DMF [ATV in 10% DMF (40)] or ATV-NE#6 with 10, 20, and 40 mg/kg of ATV [ATV-NE#6 (10), ATV-NE#6 (20), and ATV-NE#6 (40), respectively] for 21 days. (A) Tumor growth inhibition in each group. (B) Photographs of tumors from each group excised on day 21 (Scale bar: 10 mm). (C) Isolated tumor weights in 4T1-cell-bearing mice. (D) Changes in the body weights of mice during treatment. Values are means ± SEM (*n* = 10/group). ^**^p <.01, ^***^p <.001 compared to untreated controls; ^#^*p* <.05, ^###^*p* <.001 compared to ATV in 10% DMF (40); ^$$$^*p* <.001 compared to ATV-IP (5); ^@@@^*p* <.001 compared to ATV-IP (10); ^&&&^*p* <.001 compared to ATV-NE#6 (10); ^%%%^*p* <.001 compared to ATV-NE#6 (20).

Next, tumor growth inhibition by orally administered ATV-NE#6 was investigated in the murine breast cancer model (4T1 cells). The untreated control group had continuous and rapid tumor growth with a mean of 3956 ± 325 mm^3^ by day 21 (Figure 8(A)). Compared to untreated controls, daily oral administration of 40 mg/kg of ATV in 10% DMF did not inhibit tumor growth. However, once-daily IP administration of ATV-IP (5) and ATV-IP (10) inhibited tumor growth in a dose-dependent manner by 30.4% and 52.5%, respectively, compared to controls. Meanwhile, oral administration of ATV-NE#6 for 21 days significantly suppressed the tumor volume in a dose dependent manner. ATV-NE#6 (10) and ATV-NE#6 (20) had similar tumor suppressive efficacies of 37.8% and 35.5%, respectively, compared to controls. A 40 mg/kg dose of ATV-NE#6 maximally inhibited tumor growth by 59.0% and 52.4% compared to the control and ATV in 10% DMF (40) groups, respectively. This antitumor efficacy was comparable to that of ATV-IP (10). Moreover, oral and IP administration of ATV-NE#6 (40) and ATV-IP (10) also resulted in isolated tumor mass reductions of 83.0% and 73.8%, respectively, compared to controls (Figure 8(B,C)). Oral ATV-NE#6 (10, 20, and 40) treatment did not affect the body weights of mice, indicating a lack of systemic toxicity. However, further toxicity studies following long-term oral ATV-NE administration are required (Figure 8(D)). These findings correlated well with the systemic drug levels because the oral bioavailability of ATV-NE#6 was estimated at 33% of that after IP administration. The maintenance of pharmacologically active plasma concentrations during oral ATV-NE#6 (40) therapy may directly and indirectly affect tumor cell proliferation and their microenvironments by inducing apoptosis and preventing neovascularization via mevalonate pathway inhibition (Yang et al., 2010; Kang et al., 2014; Sheng et al., 2020).

These findings indicate that chronic oral ATV-NE could be used as a well-tolerated chemotherapeutic agent to prevent and treat various cancers (including breast cancer), by increasing statin accumulation in both plasma and tumor tissues to a level that reduces the risk of cancer development and recurrence at a dose attainable in clinical practice. However, further mechanistic investigations of the anticancer properties of ATV-NE and efficacy of ATV-NE combined with other cancer therapies are required.

## 4. Conclusions

We designed a multivalent intestinal transporter-targeting ATV-loaded nanoemulsive system (ATV-NEs) by physical anchoring of TPGS, DOCA-TAP, and Biotinyl PE. The *P_app_* value of the optimal formulation (ATV-NE#6) showed significant enhancement by 11.4-fold, compared with free ATV. Investigation of intestinal transport mechanisms revealed that the inhibition of ASBT or SMVT in Caco-2/HT-29 MTX E12 cell monolayers significantly reduced the *P_app_*, suggesting that ASBT- and SMVT-mediated transport are predominant pathways for improving the oral absorption of ATV from ATV-NEs. Furthermore, the suppression of P-gp-mediated efflux by TPGS in ATV-NE#6, as well as endocytosis and macropinocytosis, actively contributed to the transport of ATV-NEs. Thus, the oral bioavailability of ATV-NE#6 (equivalent to 40 mg/kg ATV) in rats was enhanced by 11.9-fold, compared with free ATV. Moreover, the enhanced oral absorption of ATV-NE#6 (40) provided the maximum suppression of tumor growth in 4T1 tumor-bearing mice by 59.0% and 52.4% compared with the oral ATV and untreated control groups, respectively; these results were equivalent to ATV-IP (10) in terms of anticancer effects. Overall, these observations suggest that ATV-NE#6 can improve oral chemotherapy by enhancing the oral delivery of ATV.

## Supplementary Material

Supplemental Material

## Data Availability

Not Applicable
