# Supplementary material for: Enhancement of the anticancer effect of atorvastatin-loaded nanoemulsions by improving oral absorption via multivalent intestinal transporter-targeting lipids
Source: Drug Deliv. 2022 Nov 23;29(1):3397–413. doi: 10.1080/10717544.2022.2149896 (PMC9704079; doi:10.1080/10717544.2022.2149896)
Supplement: Supplemental Material [file IDRD_A_2149896_SM1394.docx]

**Supporting Information**

**Enhancement of the anticancer effect of atorvastatin-loaded nanoemulsions by improving oral absorption via multivalent intestinal transporter-targeting lipids**

Laxman Subedi^a^*, Prashant Pandey^a^*, Bikram Khadka^a^, Jung-Hyun Shim^a,b^, Seung-Sik Cho^a,b^, Seho Kweon^c^, Youngro Byun^c,d^, Ki-Taek Kim^a,b^ and Jin Woo Park^a,b^

^a^Department of Biomedicine, Health & Life Convergence Sciences, BK21 Four, Biomedical and Healthcare Research Institute, Mokpo National University, Jeonnam 58554 Republic of Korea

^b^College of Pharmacy and Natural Medicine Research Institute, Mokpo National University, Jeonnam 58554, Republic of Korea

^c^Department of Molecular Medicine and Biopharmaceutical Science, Graduate School of Convergence Science and Technology, College of Pharmacy, Seoul National University, Seoul 08826, Republic of Korea

^d^Research Institute of Pharmaceutical Sciences, College of Pharmacy, Seoul National University, Seoul 08826, Republic of Korea

* Laxman Subedi and Prashant Pandey contributed equally to this work as first authors.

**Addresses for correspondences:** Ki-Taek Kim, E-mail: [ktkim0628@mnu.ac.kr](mailto:ktkim0628@mnu.ac.kr), Department of Pharmacy, College of Pharmacy, Mokpo National University, 1666 Youngsan-ro, Muan-gun, Jeonnam 58554, Republic of Korea; Jin Woo Park, E-mail: [jwpark@mokpo.ac.kr](mailto:jwpark@mokpo.ac.kr), Department of Pharmacy, College of Pharmacy, Mokpo National University, 1666 Youngsan-ro, Muan-gun, Jeonnam 58554, Republic of Korea

**Running head:** Enhanced oral absorption of atorvastatin for chemotherapy

**Table S1.** Inhibitors and concentrations used in the transport study, listed with their functions.

| Inhibitor | Concentration | Function |
| --- | --- | --- |
| Act D | 3.2 μM | Inhibitor of ASBT-mediated transport |
| CFZ | 10 μM | Inhibitor of OST_α/β_ blocking the transport of bile acid across the basolateral membrane |
| PA | 0.2 mM | Inhibition of SMVT-mediated transport |
| Chlorpromazine | 32 μM | Inhibitor of clathrin-mediated endocytosis |
| MBCD | 10 mM | Inhibitor of caveola/lipid raft-mediated endocytosis (cholesterol depletion) |
| Genistein | 0.1 mM | Inhibitor of caveola/lipid raft-mediated endocytosis (broad inhibitor of protein tyrosine kinase) |
| Amiloride | 0.1 mM | Inhibitor of macropinocytosis |
| Brefeldin A | 90 μM | Inhibitor of ER/Golgi pathway |
| Cys A | 10 µM | Inhibitor of P-gp-mediated efflux |

Act D, actinomycin D; ASBT, apical sodium-dependent bile acid transporter; CFZ, clofazimine; OST_α/β_, organic solute transporter α and β; PA, pantothenic acid; SMVT, sodium-dependent multivitamin transporter; MBCD, methyl-β-cyclodextrin; ER, endoplasmic reticulum; Cys A, cyclosporine A; P-gp, P-glycoprotein.
